# PROTOCOL: Case management interventions seeking to counter radicalisation to violence: A systematic review of tools and approaches

**DOI:** 10.1002/cl2.1301

**Published:** 2023-02-05

**Authors:** James Lewis, Sarah Marsden, Adrian Cherney, Martine Zeuthen, Jocelyn J. Bélanger, Anastasiia Zubareva, Jürgen Brandsch, Mauro Lubrano

**Affiliations:** ^1^ Handa Centre for the Study of Terrorism and Political Violence (CSTPV), Department of International Relations University of St Andrews St Andrews UK; ^2^ School of Social Science, Faculty of Humanities and Social Sciences University of Queensland Queensland Brisbane Australia; ^3^ Terrorism and Conflict Research Group Royal United Services Institute (RUSI) Mombasa Kenya; ^4^ Department of Psychology New York University Abu Dhabi Abu Dhabi UAE; ^5^ Department of Psychology University of Limerick Limerick Ireland; ^6^ Independent Researcher Bad Schwalbach Germany; ^7^ Department of Politics, Languages & International Studies University of Bath Bath UK

## Abstract

This systematic review consists of two parts. Part I seeks to synthesise evidence from primary or secondary research studies examining the implementation and effectiveness of case management tools and approaches currently being used to counter radicalisation to violence. Part II is an ‘overview of reviews’ that seeks to identify relevant and transferable lessons from systematic reviews and meta‐analyses of case management tools and approaches used in the broader field of violence prevention that could be applied to counter‐radicalisation practice.

## 1 BACKGROUND

This review seeks to identify key lessons for researchers, policymakers and practitioners working on the problem of radicalisation to violence by examining the effectiveness of case management tools and approaches being used in this space. It aims to go further than standard Campbell systematic reviews of intervention effectiveness by incorporating two distinct parts. Part I aligns with a standard Campbell systematic review procedure by examining case management tools and approaches that are used to counter the specific problem of radicalisation to violence. Part II goes further by examining case management tools and approaches that are used to counter other forms of interpersonal and collective violence, and discussing how evidence drawn from this research might inform interventions seeking to counter radicalisation to violence. By examining research that would fall outside the scope of a review solely focused on the problem of radicalisation to violence, Part II aims to capture insights relevant to policy, practice and research that would otherwise be absent.

### 1.1 The problem

#### 1.1.1 Radicalisation to violence

Since the start of the 21st Century, the concept of ‘radicalisation’ has been used by policy‐makers and academics to refer to a variety of different phenomena, including the adoption of radical beliefs (both violent and non‐violent), as well as engagement in violent extremist and terrorist activity (Neumann, 2013). This review focuses on radicalisation to *violence*, defined as the process by which ‘a person or group takes on extreme ideas and begins to think they should use violence to support or advance their ideas or beliefs’ (Government of Canada, 2018, p. 7). The term ‘radicalisation’ is therefore used in this review to refer to the process by which individuals become engaged in or supportive of *violent* extremism, which is defined here as ‘the beliefs and actions of people who support or use violence to achieve extreme ideological, religious or political goals’ (Government of Canada, 2018, p. 7). Research examining broader forms of radicalisation (i.e., the adoption of non‐violent ‘radical’ or ‘extreme’ beliefs) is not included in this review on the basis that holding such beliefs is ‘not always illegal or necessarily problematic in and of itself’ (Government of Canada, 2018, p. 7; also Schmid, 2014).

Radicalisation is a contested concept, with some authors challenging the empirical evidence on which dominant models of radicalisation are based (e.g., Githens‐Mazer & Lambert, 2010; Kundnani, 2012). However, the term is now ubiquitous within counter‐terrorism policy circles around the world, and academic research into radicalisation has grown exponentially (Heath‐Kelly et al., 2015; Silva, 2018). This research has shown that there is no single process of radicalisation, nor a common profile of individuals who become radicalised (Horgan, 2008). Instead, individual trajectories into (and out of) violent extremism are shaped by a complex intersection between different ‘push’ and ‘pull’ factors specific to individuals and/or the contexts in which they live (Lewis & Marsden, 2021; Vergani et al., 2020; Wolfowicz et al., 2020). Interventions working with clients who are considered to be ‘at‐risk’ of radicalisation and/or those who are already involved in violent extremism must therefore be responsive to the specific and varied characteristics, circumstances and needs of each individual (Cherney, 2021; Cherney, Belton, & Koehler, 2020; Gielen, 2018; Koehler, 2017).

Interventions seeking to counter radicalisation to violence are increasingly drawing on case management approaches to deliver this type of individually tailored work to clients (Cherney & Belton, 2021a). Case management interventions, broadly defined as programmes that work with individual clients by tailoring intervention plans to each client's specific needs, have long been used to deliver client‐focused interventions in a range of other policy areas, including healthcare, social work and corrections (Lukersmith et al., 2016). Importantly, interventions incorporating a case management component have been effective in countering individual involvement in different forms of violence (e.g., Brantingham et al., 2021; Engel et al., 2013), including ideologically‐motivated violence (Weine et al., 2021). This evidence of effectiveness, coupled with research pointing to the individualised nature of radicalisation, would suggest that using case management models to deliver counter‐radicalisation work is theoretically sound, provided that the tools and approaches used are effective (Cherney, Belton, & Koehler, 2020; Koehler, 2017). However, there is a lack of clarity around the types of case management tools and approaches that are being used to counter radicalisation to violence, and their impact remains unclear.

Previous reviews have illustrated that the evidence base underpinning counter‐radicalisation interventions, including those using case management tools and approaches, is weak (Bellasio et al., 2018; Feddes & Gallucci, 2015; Pistone et al., 2019). Existing systematic reviews of programme evaluations have illustrated the paucity of studies that would meet the inclusion criteria for a standard Campbell systematic review (e.g., Pistone et al., 2019; van Hemert et al., 2014). This is, in part, because of the significant methodological and conceptual challenges facing efforts to evaluate these interventions (Baruch et al., 2018; Lewis et al., 2020). A particular challenge relates to identifying appropriate client‐level outcome measures or indicators. These are not always explicit and can vary according to the specific objectives of the intervention in question (Cherney et al., 2018), and across different clients (Gielen, 2018).

More fundamentally, no study has yet sought to systematically identify and assess the tools and approaches used in case management counter‐radicalisation interventions and understand how these are used in practice. Existing research has tended to focus on individual aspects of a case management process, with a heavy emphasis on risk assessment (Scarcella et al., 2016), and efforts to interpret outcomes (Cherney & Belton, 2020). Similarly, although a small number of studies have explored how case managed counter‐radicalisation interventions are being implemented (e.g., Cherney & Belton, 2021a; Harris‐Hogan, 2020; Weeks, 2018), there has been no systematic research that looks across the different stages of the case management process in the field of countering radicalisation to violence, and limited comparative work mapping the ways that different tools and approaches are used in practice.

Part I of this review sets out to address these key gaps in research on counter‐radicalisation. Part II aims to go further than a standard Campbell systematic review by analysing case management tools and approaches that are not specifically designed to tackle the problem of radicalisation to violence, but which might produce relevant lessons to work in this space.

#### 1.1.2 The relationship between violent extremism and other forms of violence

Violent extremism is a particular phenomenon that has characteristics that distinguish it from other forms of violence. However, it is now increasingly recognised that lessons from interventions seeking to tackle other forms of violence, such as gang‐related violence or larger‐scale militancy, could be applied to interventions seeking to counter radicalisation to violence (e.g., Baruch et al., 2018; Davies et al., 2017; Ris & Ernstorfer, 2017). Although less well‐established, there is also a growing interest in exploring how interventions working with convicted sex offenders and violent offenders might be applied to counter‐radicalisation research and practice (e.g., Cherney et al., 2021). Such lessons are likely to be particularly relevant for those seeking to design, use and evaluate case management tools and approaches in the field of counter‐radicalisation. This is because, as noted above, case management has long been used to prevent individuals from participating in other forms of targeted violence, and to tackle recidivism among violent offenders. In turn, a number of systematic reviews have examined the effectiveness of case management tools and approaches in countering violent offending (e.g., Viljoen et al., 2018). However, there has yet to be any systematic analysis of the transferability of these tools and approaches to efforts at countering radicalisation to violence. Part II of this review will seek to address this gap.

### 1.2 The intervention

Parts I and II of the review examine interventions from different fields. However, whilst these interventions seek to tackle different phenomena, namely radicalisation to violence (Part I) and other forms of targeted violence (Part II), there are links between them. This section therefore discusses both types of intervention in turn, as well as the synergies between them.

#### 1.2.1 Defining ‘radicalisation to violence’

Despite the term's ubiquity today, there remains a lack of consensus over how to define ‘radicalisation’, or how to model the radicalisation process (Silva, 2018). This has resulted in governments and their partners around the world introducing a diverse range of interventions seeking to counter radicalisation to violence that are underpinned by different approaches to prevention, different theories of change, and different models of radicalisation (Hardy, 2018).

There is an important distinction between ‘cognitive’ and ‘behavioural’ models of radicalisation (Neumann, 2013; Vergani et al., 2020; Wolfowicz et al., 2021). Whilst cognitive approaches understand radicalisation as the process by which individuals adopt extremist *beliefs*, behavioural models frame radicalisation as the process by which individuals become involved in violent *behaviour*. Interventions underpinned by the former model of radicalisation typically have a broader focus than those that are more explicitly focused on preventing violent behaviour given that a broad range of beliefs—including beliefs that are not explicitly violent—have been defined as ‘extremist’ by some governments (Schmid, 2014).

Whilst the precise nature of the causal relationship between extremist beliefs and violent action is unclear (Borum, 2012), both cognitive and behavioural models frame radicalisation as the process by which individuals transition closer towards violent action. Within cognitive models, the adoption of extremist beliefs is seen to increase the risk of an individual becoming involved in violence (Schmid, 2014), whilst involvement in violence is the end‐point of behavioural models (Neumann, 2013). In turn, interventions seeking to counter radicalisation to violence include programmes that are designed to counter violent extremist beliefs as well as those that are more explicitly focused on countering violent behaviour (Hardy, 2018; Neumann, 2013; Schmid, 2014; Sedgwick, 2010). This review will therefore consider case management interventions underpinned by both cognitive and behavioural models of radicalisation to *violence* that seek to a) tackle risk factors believed to contribute to cognitive and/or behavioural radicalisation; and/or b) strengthen protective factors believed to mitigate against both forms of radicalisation (see Wolfowicz et al., 2021). Interventions that seek to tackle broader forms of cognitive radicalisation (i.e., the adoption of non‐violent ‘extreme’ beliefs) will not be included, unless this work is explicitly linked to the prevention of violence.

#### 1.2.2 Interventions seeking to counter radicalisation to violence

The terms ‘PVE’ (preventing violent extremism) and ‘CVE’ (countering violent extremism) are often used to describe the increasingly diverse range of interventions that are designed to counter radicalisation to violence. The P/CVE field is complex, with a range of governmental and non‐governmental actors now delivering a variety of interventions. These are often categorised using the public health model of primary, secondary and tertiary prevention (e.g., Bhui & Jones, 2017; Marsden et al., 2017). In practice, the distinction between these different stages of prevention is often not absolute, and individual P/CVE interventions may span multiple stages (Cherney & Belton, 2021a). However, the distinction between primary, secondary and tertiary prevention is useful for distinguishing between different elements of P/CVE programming.

Primary interventions are ‘broad‐based, mass prevention programmes that target the general population’ to build ‘individual and communal ‘resilience’ against radicalisation’ (Elshimi, 2020, p. 234). A prominent example of primary prevention is the delivery of whole‐school educational initiatives to build pupils' awareness and understanding of the risk of radicalisation (Sjøen & Jore, 2019; Wallner, 2020). Several of these interventions have reported positive results against intended outcomes (e.g., Parker & Lindekilde, 2020). However, whilst primary interventions are crucial to a ‘whole of society’ approach to countering radicalisation, the fact that they operate in a ‘pre‐risk stage’ (Elshimi, 2020, p. 229) means that the relationship between their outcomes and individual involvement in violent extremism is not always clear. Primary interventions are therefore not included in this review.

Secondary and tertiary interventions are more explicitly focused on preventing individuals from radicalising towards and/or engaging in violence. Secondary interventions work with ‘at risk’ individuals or groups to ‘prevent the progression of radicalisation and reduce the potential for future radicalisation’ (Elshimi, 2020, p. 235). Tertiary interventions work with those already engaged in violent extremism to facilitate disengagement processes and desistance from violence, which might include engaging with family members and relevant persons from within the social network of radicalised individuals (Elshimi, 2020). It is in the secondary and tertiary prevention space that case management tools and approaches are becoming increasingly common, with prominent examples of case‐managed counter‐radicalisation interventions and programmes including Channel (HMG, 2020) and the Desistance and Disengagement Programme (DDP) in the UK (Elshimi, 2020), and the Countering Violent Extremism Early Intervention Program (CVE‐EIP) (Harris‐Hogan, 2020) and Proactive Integrated Support Model (PRISM) (Cherney & Belton, 2021a) in Australia. Given their explicit focus on delivering outcomes relevant to countering radicalisation to violence, this review defines secondary and tertiary interventions operating in this context as ‘counter‐radicalisation interventions’, and avoids using the term P/CVE for the sake of clarity.

Part I of the review will examine case management counter‐radicalisation interventions, and/or their associated tools and approaches, as defined in Section 1.2.4–6 below. This might include evidence from standalone case management interventions such as Channel or PRISM, as well as evidence from more ‘comprehensive’ programmes that utilise a case management component as part of a broader suite of different interventions (see Hodgkinson et al., 2009).

#### 1.2.3 Defining ‘Violence’

This review defines violence as ‘[t]he intentional use of physical force or power, threatened or actual’ that ‘either results in or has a high likelihood of resulting in injury, death, psychological harm, mal‐development, or deprivation’ (Dahlberg & Krug, 2002, p. 5). Violence can be categorised as either collective; interpersonal; or self‐directed (Dahlberg & Krug, 2002). Part II of the review focuses on interventions seeking to tackle collective and interpersonal violence which, as noted in Section 1.1.2, are increasingly seen as being transferable to counter‐radicalisation work. Interventions for self‐directed violence will be excluded. The review adopts the following definitions of collective and interpersonal violence:

a. Collective Violence: Physical, psychological or sexual violence perpetrated by those acting as part of a collective such as gang‐related violence (e.g., Randhawa‐Horne et al., 2019) or larger‐scale militancy (e.g., USAID, 2021).
b. Interpersonal Violence: Physical, psychological or sexual violence perpetrated by individuals (or small groups of individuals) against other people (Mercy et al., 2017), including family members or partners (e.g., Gondolf, 2008).

Secondary and tertiary interventions seeking to tackle collective (e.g., Brantingham et al., 2021; Engel et al., 2013) and interpersonal (e.g., Gondolf, 2008) forms of violence are also increasingly using case management tools and approaches in work with clients. The obvious relevance of research examining these interventions for policymakers and practitioners working to counter radicalisation to violence is illustrated by the fact that case management interventions seeking to counter violence more broadly have been used to tackle ideologically‐motivated violence (Weine et al., 2021), and have been used as a foundation for developing interventions seeking to counter radicalisation to violence (Weine et al., 2018).

In line with Part I, Part II will consider evidence from secondary and tertiary case management interventions, and their related tools and approaches, seeking to prevent individuals from engaging in, or promote disengagement/desistance from any type of violence, including but not limited to phenomena such as domestic violence, gang‐related violence, and militancy.

#### 1.2.4 The case management process

**Figure 1:**
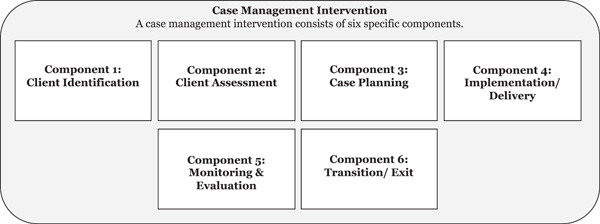
The intensive case management process (based on NCMN, 2009).

Case management interventions range from more light‐touch and short‐term ‘brokerage’ models, in which a broker connects clients with different forms of support, through to more ‘intensive’ or ‘assertive’ approaches in which case managers take a more prolonged role in supporting their clients (Lukersmith et al., 2016). Parts I and II of the review will include interventions that use any model of case management. However, as research would suggest that case management interventions seeking to counter radicalisation to violence predominantly use more intensive models of case management (e.g., Cherney & Belton, 2021a; HMG, 2020), it is expected that the most relevant lessons from the broader field of violence prevention (i.e., interventions included in Stage II of the review) will be drawn from case management interventions that use similarly intensive models as outlined in Figure 1.

The intensive approach to case management is often understood as comprising several components (see Figure 1) including client identification; client assessment; case planning; care implementation/co‐ordination; evaluation and monitoring; and transition/exit (NCMN, 2009; Ross et al., 2011). Whilst the specific terminology and exact definitions used by different organisations can vary, these components are evident in guidance produced by professional bodies such as the Case Management Society UK (CMSUK) and Canada's National Case Management Network (NCMN) (CMSUK, 2009; NCMN, 2009). A defining feature of this approach is that it is client‐centred, as shown in the definitions below:

[Case management is] a collaborative process which assesses, plans, implements, coordinates, monitors and evaluates the options and services required to meet an individual's health, care, educational and employment needs, using communication and available resources to promote quality cost effective outcomes.

(CMSUK, 2009, p. 8)

Case Management is a collaborative, client‐driven process for the provision of quality health and support services through the effective and efficient use of resources. Case Management supports the clients’ achievement of safe, realistic, and reasonable goals within a complex health, social, and fiscal environment.

(NCMN, 2009, p. 7)

Critics of case management have argued that this approach ‘places an untoward emphasis on ‘managing’ cases, addressing outcomes without a commitment to process and relentless attention to cost‐effectiveness’ (Gursansky et al., 2020, p. 4). However, the fundamental principle of case management outlined in the above definitions—that each individual client is provided with a co‐ordinated package of support that is tailored to their specific needs and circumstances (Gursansky et al., 2020)—appears well‐suited to efforts to prevent, interrupt and counter radicalisation to violence (Zeuthen, 2021).

A staged case management process should provide a foundation for: identifying the needs of individual clients and for setting client goals (assessment); for developing and delivering work plans tailored to these needs (planning and implementation); for measuring and monitoring a client's progress (evaluating and monitoring); and for assessing whether the work plan has effectively met the client's needs or delivered specific client‐level outcome measures (transition). Research would suggest that case management interventions in this field are commonly designed to work in this way (e.g., Cherney & Belton, 2021a; Weeks, 2018), but a variety of case management tools and approaches may be used across different programmes.

This review uses the terms *tools* and *approaches* to examine two distinct, but related, elements of case management interventions. These concepts are distinguished as follows:

– The term *tools* is used to refer to the procedures or methods that are used to identify individuals eligible for interventions; assess their treatment needs; develop and deliver intervention plans; monitor and evaluate their progress and treatment outcomes; and determine when the client is eligible to exit the intervention.
– The term *approaches* is used in the same way as in the White et al. (2021) evidence and gap map of interventions seeking to prevent children getting involved in violence to refer to the different theories of change or intervention logics that underpin case management interventions, whether implicit or explicit.

#### 1.2.5 Case management tools

A range of tools may be used across different stages of the case management process, and to capture client‐level progress and outcome measures (Cherney, Belton, & Koehler, 2020). Examples of three such tools are discussed below.

##### Risk assessment tools

There is a robust body of research that has examined various risk assessment tools currently being used to assess an individual's risk of violence (see Hurducas et al., 2014; Scarcella et al., 2016). These include tools that have been specifically developed for use in case management interventions seeking to counter radicalisation to violence (see Lloyd, 2019). Prominent examples include the Extremism Risk Guidance 22+ (ERG22+) (Lloyd & Dean, 2015); the Violent Extremism Risk Assessment Version 2 Revised (VERA‐2R) (Pressman, 2009); and the Terrorist Radicalization Assessment Protocol (TRAP‐18) (Meloy et al., 2015).

The primary function of these tools is the initial assessment of clients. However, there is considerable variation in the number and types of indicators included in different tools. For example, Lloyd (2019) notes that several tools explicitly capture protective factors alongside risk factors (e.g., VERA‐2R), whilst others only focus on risk factors. There are also some tools that assess protective factors only (e.g., Abbiati et al., 2020). This means that the specific assessment tool used may influence the types and range of client needs identified and targeted.

Many of these risk assessment tools are used across multiple stages of the case management process. They may be used to develop case plans to mitigate risk factors and/or strengthen protective factors identified in initial client assessments, to monitor clients and assess client‐level progress, and to determine when clients are eligible to exit the intervention (Lloyd & Dean, 2015; Lloyd, 2019). For example, official guidance for the UK's Channel programme states that the same risk assessment tool—the Vulnerability Assessment Framework (VAF), which is based on the ERG 22+ tool mentioned above (Augestad, 2020)—should be used across all stages of the case management process. Each client has a ‘Channel Case Officer’ who is responsible for ‘updating the VAF and for assessing [client] progress’, with the expectation being that the VAF will be updated every three months as a minimum ‘to ensure that the progress being made in supporting the individual is being captured’ (HMG, 2020, p. 36). The VAF should also be reassessed when it is needed ‘to inform a key panel meeting’; ‘where the provision of support has reached a particular milestone’, or where there have been ‘significant changes to circumstances or levels of risk’ (HMG, 2020, p. 36). If the Case Officer and a multi‐agency panel is satisfied that an individual's level of risk has been sufficiently reduced upon completion of their work plan, the case will be closed, and a final VAF will be completed.

##### Case planning tools

Whilst several of the risk assessment tools listed above are explicitly designed to be used to inform case planning (Lloyd, 2019), exploratory research from other fields has also suggested that dedicated case planning tools could be used to ensure that case plans accurately reflect and target the issues identified by assessments tools (Viljoen et al., 2018, 2019). There has been little research into the use of case planning tools in the field of countering radicalisation to violence or related phenomena. However, insights from the wider field of violent offending highlights the limitations of relying solely on data collected from risk assessment tools when developing work plans (Viljoen et al., 2018, 2019). Whilst risk assessment tools can be used throughout the case management process, additional data will likely be needed to ensure that interventions are delivered in ways that are consistent with their own internal logic; that intervention plans are appropriate for targeting the risk/protective factors identified by risk assessments; and that the support delivered to each individual clients is in turn consistent with their original intervention plan. These are all issues that could be addressed by using case planning tools.

##### Monitoring and evaluation tools

Potential sources of monitoring and evaluation data include administrative data relating to a client's participation in an intervention (Cherney, Belton, & Koehler, 2020), or information about a client's activities outside of the intervention gleaned from family members or peers (Cherney, 2021; Meloy, 2018). Case notes are likely to be a particularly important data collection and analysis tool (Cherney & Belton, 2021a; Cherney, Belton, & Koehler, 2020; Meloy, 2018). Case notes may be compiled using pre‐defined templates or may be more ad hoc (Cherney, Belton, & Koehler, 2020), and completed by one or multiple practitioners throughout an intervention (Cherney & Belton, 2021a). Case notes containing an appropriate level of detail will include information which can be used to identify client‐level progress and outcome measures, and to assess progress against said measures (Cherney & Belton, 2021a). As Cherney, Belton, and Koehler (2020, p. 23) outline, this might include ‘risk assessment data, criminal history (if applicable), and detailed information of client radicalisation (including triggering events); treatment plans and intervention goals and how clients are progressing towards these goals (milestones achieved); and contact and interactions with clients’. In summary, a diverse range of tools may be used across different stages of the case management process, and more research is needed to understand how these tools are being used in practice.

#### 1.2.6 Case management approaches

Researchers often distinguish between different counter‐radicalisation ‘approaches’ that are underpinned by different theories of change. Most notably, a distinction is often drawn between ‘strengths‐based’ and ‘risk‐oriented’ approaches to countering radicalisation (e.g., Marsden, 2017). These are informed by different assumptions about how best to counter radicalisation; both approaches are discussed in the analysis of mechanisms below.

In many cases, the assumptions, or theory of change, underpinning interventions seeking to counter radicalisation to violence are not explicit (Lewis et al., 2020). This means that identifying and examining different approaches to case management can be challenging. However, it is possible to identify and map the different approaches being used to counter radicalisation to violence by examining the constituent parts of each intervention's theory of change—implicit or explicit—and to synthesise evidence relating to interventions underpinned by similar assumptions.

**Figure 2:**
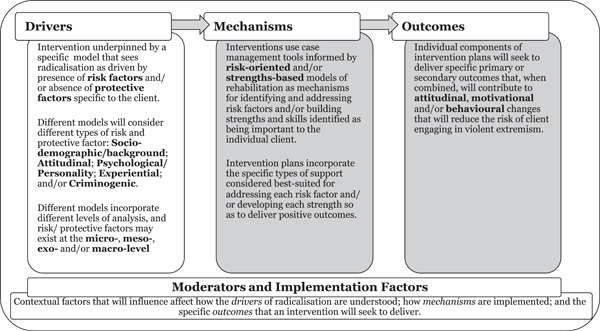
Case management intervention theory of change.

All interventions seeking to counter violence are underpinned by implicit or explicit assumptions about the drivers of violence, and how violence is best prevented (Cherney, Belton, & Koehler, 2020). These assumptions can be identified by breaking down an intervention's theory of change into its constituent parts. Cherney, Belton, and Koehler (2020, pp. 12–13) note that ‘developing a theory of change requires consideration of what will be targeted (which drivers of violent extremism) and how they will be targeted (mechanisms) to achieve what (outcomes), which will be influenced by certain factors (moderators and implementation processes)’. Different approaches to case management can be categorised using the four domains of *drivers*, *mechanisms*, *outcomes* and *moderators* (Figure 2 below).

The sub‐sections below draw on existing research on radicalisation and counter‐radicalisation and discuss how different approaches to case management can be identified by unpicking the constituent parts of their theories of change. Whilst the discussion below is oriented towards Part I of the review, and therefore focuses on case management interventions seeking to counter radicalisation to violence, it is illustrative of how different approaches to case management more broadly (i.e., including those examined in Part II) can be differentiated.

**Table 1:** Interventions and drivers of radicalisation.

Primary focus of Intervention	Criteria
Risk factors	Intervention and its associated tools only focus on identifying and tackling risk factors, or pay limited attention to protective factors.
Protective factors	Intervention genuinely integrates protective factors and resilience‐building activities.

##### Case management approaches I: Drivers

Radicalisation is commonly understood as a process that is driven by the presence of risk factors and/or the absence of protective factors, with different models of radicalisation placing different weight on the relative importance of risk versus protective factors (see Vergani et al., 2020; Wolfowicz et al., 2021). Interventions seeking to counter radicalisation to violence often consider both risk and protective factors (Dean, 2014). However, there may be variation in how explicitly an intervention (and associated tools) focuses on the latter (Table 1). Interventions can be interpreted in relation to the relative weight they place on risk and protective factors throughout the different stages of the case management process outlined in Figure 2.

###### Different types of risk/protective factor that an intervention might focus on

The relationship between risk and protective factors is complex and contested (Lösel et al., 2018). However, it is common to interpret them thematically. For example, Wolfowicz et al. (2021) Campbell systematic review of risk and protective factors for cognitive and behavioural radicalisation categorised risk and protective factors across five domains (see Table 2 below). Whilst it might be expected that interventions would consider multiple domains, the specific focus of each intervention may vary according to the model of radicalisation that underpins it (Hardy, 2018), or the broader theories of (violent) human behaviour that underpin violence prevention programmes, and in the context of case management, the needs of each client.

**Table 2:** Different domains (Wolfowicz et al., 2021).

Domain	Examples
**Socio‐demographic/background:** Individual characteristics that might contribute to, or mitigate against, radicalisation risk.^a^	**Risk Factors**
	Unemployment, alcohol or substance abuse, relationship problems etc.
	**Protective Factors**
	Socio‐economic status, education etc.
**Attitudinal factors**	**Risk Factor**
Holding specific attitudes or perceptions about the world that can contribute to, or mitigate against, radicalisation risk.	Perceived in‐group superiority, perceived discrimination, perceived injustice etc.
	**Protective Factors**
	Law abidance, belief in legitimacy of the law, trust in institutions, trust in others, social support, perceived self‐efficacy etc.
**Psychological/Personality factors**	**Risk Factors**
Psychological or personality traits that can contribute to, or mitigate against, radicalisation risk.	Mental health issues, anger, negative affect, authoritarianism etc.
	**Protective Factors**
	Life satisfaction, higher self‐esteem etc.
**Experiential**	**Risk Factors**
Life experiences that can contribute to, or mitigate against, radicalisation risk.	Prior incarceration, experience of discrimination, traumatic experiences etc.
	**Protective Factors**
	High perceptions of procedural justice.
**Criminogenic**	**Risk Factors**
Traditional risk/protective factors cited as relevant to other types of criminal offending.	Criminal history, deviant/radical peers, low self‐control, thrill seeking etc.
	**Protective Factors**
	Parental involvement, school bonding, outgroup friends etc.

^a^ Of course, interventions are not able to change many factors captured by this domain (e.g., age, gender).

###### Risk/protective factors existing at different levels of analysis

Risk and protective factors operate at different levels of analysis. Most commonly, authors identify three levels of analysis: *individual* (or micro‐level); *social* (or meso‐level) and *structural* (or macro‐level) drivers (Cherney, Belton, & Koehler, 2020; Schmid, 2013). However, some models also distinguish between meso‐level and exo‐level drivers of radicalisation, the former referring to influences operating in an individual's immediate environment such as family or peers, and the latter to ‘more distal environmental influences such as community organizations’ (Ellis et al., 2022, p.2). Whilst some interventions continue to focus solely on the micro‐level of analysis (e.g., Muluk et al., 2020), a range of interventions—including those that using case management tools—now take a ‘socio‐ecological’ approach to understanding vulnerability and/or resilience (e.g., Ellis et al., 2022; Grossman et al., 2022). These socio‐ecological interventions explicitly consider and seek to address risk and protective factors existing in an individual's micro‐, meso‐, exo‐, and macro‐system (Ellis et al., 2022). Interventions can therefore be differentiated according to the levels of social ecology considered when assessing risk and protective factors (see Table 3).

**Table 3:** Different levels of analysis (based on Ellis et al., 2022).

Code	Examples
**Micro‐level**	Background factors such as those shown in Table 2
Individual characteristics	
**Meso‐level**	Extremist peers or family members (Kenney & Chernov Hwang, 2021); Poor familial relationships or household dysfunction (Gill et al., 2021)
Factors found in the immediate environment	
**Exo‐level**	Neighbourhood context (Neve et al., 2020)
Distal environmental factors	
**Macro‐level**	Political or societal discrimination against group (Cherney, Belton, & Koehler, 2020); Increased availability of extremist propaganda or increasing radicalism of mainstream (Ellis et al., 2022).
Larger social and cultural context	

##### Case management approaches II: Mechanisms

Mechanisms refer to the specific ways in which the drivers of radicalisation/other forms of violence will be targeted to produce desired outcomes (Cherney, Belton, & Koehler, 2020). The mechanisms underpinning interventions are characteristic of, and therefore can help identify, the different models of rehabilitation that inform the use of case management tools and the delivery of specific types of support to case‐managed clients. One of the core distinctions is between ‘risk‐oriented’ models and ‘strengths‐based’ models of rehabilitation. Each model is underpinned by different assumptions about the mechanisms that might be used to prevent engagement in, or facilitate disengagement from, violent extremism.

Counter‐radicalisation interventions have traditionally been rooted in risk‐oriented models of rehabilitation (Marsden, 2017). A risk‐oriented case management approach can be identified implicitly through the use of specific tools, such as risk assessment tools that fail to capture protective factors (see above), or explicitly through reference to specific models such as the Risk‐Needs‐Responsivity (RNR) model of case management which has long dominated in post‐conviction contexts (Bonta & Andrews, 2017). Interventions underpinned by the RNR model attempt to manage risk by tailoring intervention plans to each client's level of risk (with higher‐risk individuals receiving more intensive support), to their specific needs, in a way that is responsive to their specific characteristics and circumstances (Dean, 2014; Marsden, 2017).

The RNR approach is underpinned by a robust evidence base within the field of criminology (Bonta & Andrews, 2010), and has been used in the counter‐radicalisation context (e.g., Dean, 2014). However, researchers are increasingly calling for, and in some cases have developed, interventions that use strengths‐based approaches as mechanisms for countering radicalisation to violence (Cherney, 2021; Marsden, 2017).

Strengths‐based approaches can be distinguished implicitly through the use of tools that orient interventions towards the development of strengths or skills that are believed to contribute to rehabilitation. They can also be identified by the explicit use of strengths‐based or desistance‐informed approaches such as the Good Lives Model (GLM) which place greater emphasis on building those strengths believed to contribute to desistance (Dean, 2014).

Strengths‐based approaches seek to promote the strengths of offenders so they can satisfy their needs without resorting to violence (Marsden, 2017). Desistance‐oriented approaches focus on identifying and leveraging factors that explain why individuals desist from, or do not become engaged in, violence (Marsden, 2017). This distinction between risk‐oriented and strengths‐based approaches will make a particularly useful contribution, as it will speak directly to a long‐standing debate about the virtue of using these different approaches to tackling radicalisation and other social harms (Cherney, 2021; Horan et al., 2020; Marsden, 2017). Proponents of strengths‐based or desistance approaches argue that risk‐oriented approaches are too ‘deficit focused’ (Wong & Horan, 2021). Further, that secondary and tertiary interventions would be better served by focusing on developing clients’ internal strengths so that they are able to pursue more pro‐social ways of meeting the needs that might otherwise be served through engagement in violent (extremist) behaviour (Marsden, 2017). This review aims to contribute to this debate by examining and contrasting these different approaches.

##### Case management approaches III: Outcomes

The ultimate aim of case management interventions is the prevention of future violent extremist behaviour. However, individual components of these interventions may seek to deliver more specific outcomes that are believed to contribute to the over‐arching goal of countering radicalisation to violence. These outcomes may be informed by risk‐oriented approaches, and in turn be used to measure the effectiveness of an intervention at tackling different radical outcomes such as those set out in systematic reviews conducted by Wolfowicz et al. (2021) and Lösel et al. (2018), which focus on attitudes, motivations, and behaviours.

However, outcomes may also be strengths‐based. These are more oriented towards the development of key strengths and skills that are believed to contribute to disengagement from violent extremism, such as the development of pro‐social supports, education and training, and employment (Cherney & Belton, 2021a, 2021b). Individual components of intervention plans may seek to deliver these outcomes explicitly or implicitly. For example, case management plans may incorporate counter‐ideological or counter‐narrative work that is explicitly designed to tackle a client's violent extremist attitudes. However, they might also attempt to tackle these attitudes implicitly by seeking to address broader issues identified in the client's life believed to make them receptive to extremist ideology in the first place.

In this way, different approaches to case management can be identified by examining the primary and secondary outcomes that an individual intervention seeks to deliver. Whilst many counter‐radicalisation interventions do not explicitly adhere to the models of rehabilitation outlined in the section on mechanisms above, their implicit use of these mechanisms can be determined by assessing whether their intended outcomes align with the key assumptions underpinning RNR, strengths‐based, and desistance‐oriented frameworks.

##### Case management approaches IV: Moderator and implementation factors

Moderators refer to ‘contextual conditions’ or the ‘features of the people or places that are the target for intervention’ (Cherney, Belton, & Koehler, 2020, p. 15). The contextual conditions of case management interventions may vary in regard to, for example, the delivery context (e.g., pre‐ vs. post‐conviction settings; correctional vs. community‐based interventions, etc.); and profiles of individual clients (e.g., demographics; social context, etc.).

Implementation factors refer to ‘actions or actors necessary to successfully install and maintain an intervention’ (Cherney, Belton, & Koehler, 2020, p. 16). These factors might include the number and type of delivery agents involved in delivering an intervention; how the specific stages of case management are implemented in practice (e.g., when and how frequently are risk assessments conducted? Who conducts risk assessments? What is the duration of follow‐up etc.); and a range of contextual factors that might influence how an intervention is implemented (and in turn, its effectiveness), such as staffing levels (Wolff et al., 2022); the quality of training received by case managers/other key staff (Radcliffe et al., 2018), or the size of each case manager's caseload (Fox et al., 2022).

### 1.3 How the intervention might work

There is no single approach to delivering case management. Below is an illustrative example of how each stage of the case management process might work when engaging with clients who are at risk of becoming involved or who are already involved in violent extremism.

#### 1.3.1 Planning: Identification, assessment and case planning

In secondary interventions, ‘at‐risk’ individuals may be identified through different channels. In the UK for example, the 2015 Prevent Duty placed a range of public institutions under a statutory duty to have ‘due regard to the need to prevent people from being drawn into terrorism’ (HMG, 2021). This led to an increase in the number of individuals being referred to Channel, with referrals coming from a diverse range of sectors including healthcare, education and the police (Home Office, 2020).^1^ Whilst the UK is currently one of the only countries to legally mandate that public institutions have a means of identifying and reporting those who may be at risk of radicalisation, public sector workers in other countries perform a similar counter‐extremism function (Harris‐Hogan, 2020; Parker et al., 2021; Wallner, 2020).

A number of interventions, including Channel, use multi‐agency panels to assess individuals seen as potentially at risk of radicalisation (Ellis et al., 2022; Harris‐Hogan, 2020; HMG, 2020). A significant number of initial referrals may be screened by gatekeepers (such as local counter‐radicalisation practitioners) without going forward for formal assessment (HMG, 2020). Referrals taken forward to a multi‐agency panel will be assessed to determine whether they require a counter‐radicalisation intervention or if they need to be signposted to other forms of support. As noted above, there are a range of existing risk assessment tools that can be used to inform this decision. For example, decisions around whether to adopt an individual as a Channel case are made by local multi‐agency panels using the VAF (Pettinger, 2020).

Where an individual is assessed as in need of a counter‐radicalisation intervention, the panel designs a comprehensive work plan to address the client's specific vulnerabilities, and/or to build protective factors at the individual and/or the socio‐ecological level (e.g., Ellis et al., 2022). Work plans may be further tailored to the individual client in a number of ways. For example, case managers may decide to tailor the specific intervention providers used, or may sequence the different components of the work plan so they are delivered according to each individual client's needs or learning style (Cherney & Belton, 2021b). The specific focus of this work plan is likely to vary according to whether the intervention is underpinned by a risk‐based or strengths‐based approach, with the former focused on tackling risk factors seen as contributing to radicalisation, and the latter on building protective factors (Marsden, 2017).

Case management in tertiary interventions operates in a similar way. For example, interventions operating in post‐conviction settings use similar risk assessment tools—including those listed earlier—to assess the intervention needs of individuals who have been convicted for terrorism‐related offences, as well as those who are suspected of becoming radicalised during their incarceration. The results of these assessments inform the development of tailored intervention plans (Cherney & Belton, 2021a). One notable difference is that whilst engagement in secondary interventions tends to be on a voluntary basis, engagement in some tertiary interventions is mandatory (Elshimi, 2020), and there is some debate as to whether mandating participation is effective (Cherney et al., 2021).

Clients of both secondary and tertiary programmes are supposed to be provided with holistic intervention plans incorporating different types of support, including psychological, social, theological or ideological components (Cherney & Belton, 2021b). Whilst a number of intervention providers may be tasked with delivering these various forms of support, the delivery of individual intervention plans is typically co‐ordinated by a specific case manager, who is responsible for monitoring client‐level progress and outcomes—often with input from the intervention providers (Cherney & Belton, 2021b; Harris‐Hogan, 2020; HMG, 2020).

#### 1.3.2 Delivery: Implementation, monitoring, evaluation and transition

The theories of change underpinning case management interventions are often not explicit which means the relationships between an intervention's activities and outcomes are not always clear (Lewis et al., 2020). There can also be variation across programmes as to the individual‐, community‐, and programme‐level outcomes they are seeking to achieve (Cherney et al., 2018; Harris‐Hogan, 2020). For example, whilst some tertiary interventions seek to promote desistance and disengagement by ‘deradicalising’ clients so they reject extremist ideology (El‐Said, 2012; Khalil et al., 2019), there is some debate as to whether deradicalisation is possible or needed to promote the rehabilitation and reintegration of violent extremists (Horgan & Braddock, 2010). As a result, deradicalisation is not always an explicit objective of tertiary interventions. However, case management interventions are a specific type of ‘risk reduction initiative’ (Horgan & Braddock, 2010) as they are designed to reduce the risk of an individual becoming radicalised (secondary), or reduce the risk of an individual becoming re‐engaged in violent extremism by promoting their desistance, disengagement and/or deradicalisation (tertiary). And, as noted above, a diverse range of risk assessment tools and other data sources may be used to monitor an individual's participation in, or compliance with, an intervention plan; to monitor relevant attitudinal and behavioural changes over time; and to inform exit decisions.

### 1.4 Why it is important to do this review

Case management interventions are increasingly used by governments and their partners to prevent engagement in, and promote disengagement from, violent extremism (Cherney & Belton, 2021a). Whilst several of these programmes are long‐established, they have come under scrutiny in recent years in the wake of high‐profile terrorist attacks (Clubb et al., 2021). For example, the UK's Desistance and Disengagement Programme (DDP) (see Elshimi, 2020) attracted public attention when it became known that Usman Khan, who killed two people in a terrorist attack in 2019, had taken part in the programme. This raised questions around the extent to which intervention providers had effectively identified and addressed the specific issues present in this individual case, and the extent to which existing tools were effectively able to assess the level of risk posed by Khan upon his release from prison. The fact that Khan had been released on license also raised questions about how to monitor client progress post‐release, which is a challenge identified by practitioners in other countries (Cherney, 2021).

By incorporating two distinct, inter‐related parts, this review will make a number of important contributions that are timely to policymakers and researchers dealing with these questions.

#### 1.4.1 The importance of reviewing existing case management tools and approaches

Part I of this review will make three specific contributions. First, it will synthesise evidence from a field in which the principles of case management are often embedded in interventions, but the specific term ‘case management’ is rarely used. Bar a small number of exceptions (e.g., Cherney & Belton, 2021a), many client‐focused interventions seeking to counter radicalisation to violence do not explicitly use the term ‘case management’, which hampers evidence synthesis. By breaking down case management into its constituent parts (e.g., client identification; client assessment; care planning; implementation/co‐ordination; evaluation and monitoring; and transition/exit) Part I will identify and synthesise evidence relevant to different phases of case management, including studies that do not specifically use this term.

Second, whilst a number of empirical studies have examined the psychometric properties of risk assessment tools used in case management counter‐radicalisation interventions (see Lloyd, 2019), there has yet to be any systematic research that synthesises knowledge about how these tools are used in practice. Whilst it would be expected that validated risk assessment tools are being used to inform case planning and intervention delivery, research from other fields of violence prevention shows this is not always true in practice (Viljoen et al., 2018). This review will complement the systematic review into the psychometric validity of these tools (Hassan et al., 2022) by examining how tools are being used across the case management process to inform the development and delivery of client‐focused intervention plans, and to monitor and evaluate client progress and outcomes. This will provide a more holistic overview of how the different stages of the case management process are being operationalised within counter‐radicalisation interventions than currently exists.

Third, by synthesising evidence relating to different approaches to case management (e.g., strengths‐based approaches; risk‐oriented approaches, etc.) this review will attempt to make the implicit assumptions that underpin counter‐radicalisation interventions more explicit, and to examine the validity of different assumptions more systematically than has been done previously. This review does not examine the individual components of each intervention's theory of change (i.e., the specific change mechanism). Instead, it attempts to synthesise evidence relating to each over‐arching case management approach that is used in this space. This analysis is aligned with Hodgkinson et al.'s (2009) widely‐cited systematic review of ‘comprehensive’ anti‐gang interventions, in which the authors argued it was ‘valuable to examine the effectiveness of interventions grouped by [different] theories of change’ (p.45).

The importance of this type of analysis is illustrated by a recent systematic review of the Good Lives Model (GLM) conducted by Mallion et al. (2020), which, as noted above, is a specific approach that has been used to inform counter‐radicalisation interventions (Dean, 2014). This review set out to test the effectiveness of the GLM in the context of a range of offence types, as well as examine the assumptions on which it is based. These assumptions include the idea that all individuals are driven by a desire to live a good life, and that offending occurs when an individual faces barriers that prevent them from pursuing ‘primary goods’ that contribute to a good life—such as success in one's professional or personal life, a sense of community, etc.—in a pro‐social way. Mallion et al.'s review only included interventions explicitly stating that they were using a GLM approach, *and* which were delivered in a GLM‐consistent way (i.e., that identified primary goods relevant to the client and the barriers to pursuing them in pro‐social ways, and tailored the intervention accordingly). By synthesising evidence drawn from interventions sharing a common approach, this review was able to examine the assumptions underpinning the GLM; and the effectiveness of GLM‐informed interventions. Their review therefore provides a template for identifying different approaches by reviewing the underlying assumptions of an intervention, for assessing an approach's effectiveness in terms of impact on client‐level outcomes, and for evaluating whether an intervention is implemented in a way that is consistent with these underlying assumptions.

#### 1.4.2 The importance of learning from other fields

The limitations of the evidence base underpinning counter‐radicalisation interventions are well‐established (Baruch et al., 2018; Bellasio et al., 2018; Feddes & Gallucci, 2015; Pistone et al., 2019). It is extremely challenging to evaluate the effectiveness of these interventions given the difficulty in accessing data, the small samples of intervention participants, and the absence of an obvious counterfactual (Lewis et al., 2020). Ethical and national security considerations have also limited the use of experimental or more robust quasi‐experimental designs when evaluating secondary or tertiary interventions on the basis that it would be unethical (and potentially unsafe) to deny or delay the delivery of a counter‐radicalisation intervention to a control group simply for the purposes of evaluating it (Braddock, 2020; Lewis et al., 2020). Consequently, existing case management interventions have not been systematically evaluated, and the field lacks agreed measures to assess client‐level progress and outcomes (Cherney & Belton, 2021a). Although authors have discussed potential solutions to these evaluation challenges by drawing on lessons from other fields (e.g., Baruch et al., 2018; Davies et al., 2017; Lewis et al., 2020), the solutions proposed have yet to be applied in practice.

Part II of this review is specifically designed to address known weaknesses in the evidence‐base underpinning interventions seeking to counter radicalisation to violence. Whilst the overlap between the broader field of violence prevention and counter‐radicalisation should not be overstated, the staged counter‐radicalisation case management process outlined in Section 1.3—and the tools and approaches associated with each stage of the process—is similar to how intensive models of case management are known to operate in other fields. Given that several violence reduction interventions which incorporate a case management component have been subject to the type of robust evaluation that has often been lacking in the field of counter‐radicalisation (e.g., Brantingham et al., 2021; College of Policing, 2021a; Romaniuk & Davies, 2017; Weine et al., 2021), evidence from broader violence prevention work may provide a more robust evidence‐base with which to examine whether case management tools and approaches can be effective in countering violent attitudes and behaviours, and exploring how they might directly contribute to these types of outcomes.

By reviewing research on case management interventions seeking to tackle other forms of violence, Part II of the review seeks to identify evidence from related fields that could be used to inform interventions seeking to counter radicalisation to violence. This part of the review meets the Cochrane collaboration definition for an ‘overview of reviews’ (Pollock et al., 2021) on the basis that it will summarise evidence from systematic reviews ‘of the same intervention for different conditions or populations’ (i.e., case management interventions seeing to counter different forms of non‐extremist violence). However, Part II of the review will go further than an overview of reviews traditionally would, as it will examine the transferability of this evidence to another distinct but related field, namely countering radicalisation to violence. In this way, whilst the two parts of this review examine different types of violence and use different methodologies, they are interconnected, and will both have important implications for research, policy and practice in the field of countering radicalisation to violence on the basis that the objectives for both stages of the review are firmly orientated towards this field.

#### 1.4.3 Potential importance to policymakers and practitioners

This review speaks directly to concerns that have been raised by policymakers and by public commentators about the efficacy of case management counter‐radicalisation interventions. It does so in two ways. First, by synthesising evidence from an ever‐expanding and complex literature on counter‐radicalisation, Part I of the review aims to contribute to policy and practice by (a) examining whether the use of case management in this context is supported by evidence; (b) providing a robust assessment of the potential efficacy of case management in this context, and ‘what works, for whom, how and in what circumstances’ (Gielen, 2018); and (c) identifying areas of good practice that could be used to inform programme development. Second, by drawing on evidence from outside of the field of countering radicalisation to violence, Part II of the review aims to provide a foundation for policymakers and practitioners to learn from the use of case management in more established fields of violence prevention.

## 2 OBJECTIVES

Whilst Parts I and II of this review examine interventions seeking to counter different but related problems, both parts seek to deliver comparable analyses by (1) examining the effectiveness of case management tools and approaches in delivering primary and secondary outcomes relevant to countering violence; and (2) examining the processes by which these tools and approaches are implemented in practice. This twin focus is informed by guidance for conducting evaluations of terrorism risk reduction programmes developed by Williams and Kleinman (2014). This approach proposes that such evaluations should be ‘designed to understand both what is being done by the initiative, and how faithfully/consistently its components are implemented’ (p. 114). It is also aligned with Viljoen et al.'s systematic review into whether the use of risk assessment tools helps to reduce violence risk, which argues that:

violence reduction is not the only possible indicator of tools’ impact. If risk assessment tools do, indeed, reduce violence and offending, this relationship may be indirect, and contingent upon professionals’ risk management practices.

(Viljoen et al., 2018, p. 184)

The primary objectives of this review are therefore to synthesise:

(1) Evidence relating to the *effectiveness* of the different case management tools and approaches that are currently being used to counter radicalisation to violence.
(2) Evidence relating to the *implementation* of these tools and approaches.
(3) Identify relevant insights from wider research on interventions seeking to counter other forms of violence and assess their *transferability* to counter‐radicalisation work.

Parts I and II of the review are guided by specific research questions.

**Part I: Evaluating Case Management Counter‐Radicalisation Interventions**

**Table:** 

RQ	Question	Objective	Methodology
1	To what extent are different case management tools or approaches effective at countering radicalisation to violence?	Synthesise evidence relating to outcomes capturing the effectiveness of a tool or approach at countering radicalisation to violence. This analysis examines:	Narrative summary of all included studies.
		(i) *Primary outcomes* (i.e., measures of disengagement, desistance, and deradicalisation); and	Meta‐analysis of experimental/stronger quasi‐experimental quantitative studies.
		(ii) *Secondary outcomes* (i.e., measures of client engagement or motivation; measures capturing attitudinal or behavioural changes relevant to primary outcomes).	Alternative synthesis of data not suitable for meta‐analysis using methods Cochrane deem to be acceptable (see Section 3.8.3).
2	(a) To what extent are different case management tools or approaches implemented as intended?	Synthesise evidence that captures how different tools and approaches are being implemented in practice.	Narrative summary of all studies (qualitative and quantitative)
	(b) What explains how different case management tools and approaches are implemented in practice?	Identify, and synthesise evidence relating to, the different implementation factors and/or moderators (Section 1.2.6) that:	Meta‐analysis of quantitative studies with experimental or stronger quasi‐experimental design.
		(i) influence how different tools and approaches are implemented on‐the‐ground; and	Alternative synthesis of data not suitable for meta‐analysis using methods Cochrane deem to be acceptable (see Section 3.8.3)
		(ii) explain whether these tools and approaches are being implemented in the way intended/expected.	Framework synthesis of qualitative data.

**Part II: Learning from related fields**

**Table:** 

RQ	Question	Objective	Methodology
3	To what extent are different case management tools or approaches effective at countering interpersonal and collective forms of violence?	Synthesise evidence relating to different outcomes capturing the effectiveness of a tool or approach at countering violence. This analysis will examine both:	Narrative summary of evidence relating to each research question.
		(i) *Primary outcomes* (i.e., measures of disengagement/desistance); and	
		(ii) *Secondary outcomes* (i.e., measures of client engagement or motivation; measures capturing attitudinal or behavioural changes relevant to primary outcomes).	
4	(a) To what extent are different case management tools or approaches implemented as intended?	Synthesise evidence that captures how different tools and approaches are being implemented in practice.	
	(b) What explains how different case management tools and approaches are implemented in practice?	Identify, and synthesise evidence relating to, the different implementation factors and/or moderators (Section 1.2.6) that:	
		(i) influence how different tools and approaches are implemented on‐the‐ground; and	
		(ii) explain whether these tools and approaches are being implemented in the way intended/expected.	
5	Is there any evidence to suggest that these tools and approaches could be effective in countering radicalisation to violence?	Identify case management tools, methods and approaches used to counter other forms of violence which could potentially be adopted to counter radicalisation to violence by assessing the transferability of these interventions (see Section 4.8.2)	

Whilst there are synergies between the objectives of Part I and Part II of the review, there are important methodological differences. It is therefore necessary to outline the methodologies for each part separately. To this end, Section 3 outlines the methodology for identifying and analysing primary research studies examining the different case management tools and approaches used to counter radicalisation, before Section 4 outlines the methodology for an ‘overview of reviews’ (Pollock et al., 2021) from the broader field of violence prevention.

## 3 METHODOLOGY—PART I

### 3.1 Criteria for including and excluding studies

The inclusion criteria for research relating to questions 1 and 2 are comparable in terms of the specific *problem* (i.e., radicalisation to violence) and the type of *intervention* (i.e., case management) that will be analysed across both questions. However, as each question focuses on a different types of data (i.e., effectiveness vs. implementation), they will require separate inclusion criteria relating to the *comparator* conditions (i.e., research designs) and *outcome* measures that are relevant. This section will therefore first discuss the specific outcome measures and research designs that will be included in the analysis relating to each question, before discussing criteria that will be applied across Part I of the review as a whole.

#### 3.1.1 Outcomes

##### Outcomes relevant to countering radicalisation to violence (Question 1)

Effectiveness will be evaluated by reviewing research that reports on case management interventions (and related tools or approaches) with primary and secondary client‐level outcome measures relating to countering radicalisation to violence. To be included in Part I of the review, studies must report on the below primary and/or secondary outcomes:

– **Primary outcomes** will include measures of disengagement, desistance or deradicalisation captured using, for example, risk assessment tools; arrest or prosecutions data; case notes; and qualitative or quantitative data relating to recidivism.^2^
– *Secondary outcomes* will include data on the impacts that specific tools or approaches have on client participation/engagement with interventions (see Netto et al., 2014). A particularly important metric will be an intervention's rate of attrition, as attrition is a known issue when evaluating interventions seeking to counter radicalisation to violence (Williams, 2021). Secondary outcomes will also include data relating to risk and/or protective factors believed to be relevant to engagement in/disengagement from violent extremism, including background, attitudinal; psychological, experiential; and criminogenic factors (Wolfowicz et al., 2021). Whilst this list is not exhaustive, a range of relevant measures can be identified from risk assessment tools including:
  ○ *Extremism Risk Guidance 22+ (ERG 22+)*: Assesses risk using measures relating to three domains: *engagement* with a group, cause or ideology; *intent* to cause harm; and *capability* to cause harm (Lloyd & Dean, 2015).
  ○ *Violent Extremism Risk Assessment Version 2 Revised (VERA‐2R)*: Assesses risk against beliefs, attitudes, and ideology; history, action and capacity; commitment and motivation; protective/risk mitigating indicators; and criminal history, personal history, and mental disorder (Pressman, 2009).
  ○ *Terrorist Radicalization Assessment Protocol (TRAP‐18)*: Assesses risk across proximal warning behaviours; and distal characteristics (Meloy et al., 2015).

Evidence for the effectiveness of an intervention in delivering such outcomes may be captured in different ways. For example, client‐level impact may be assessed using tools that capture whether specific risk factors have been successfully eliminated and/or specific protective factors have been successfully developed. However, other interventions may use tools to assess a client's ‘distance travelled’ towards positive outcomes (e.g., Romaniuk & Davies, 2017).

##### Outcomes relevant to implementation (Question 2)

The process of implementation will be evaluated by reviewing evidence relating to how different tools and approaches are being used, adopted and understood in practice, and whether they are being used in ways that are consistent with their internal logic and any principles of best practice that have been developed. For example, are risk assessment tools used to inform case planning? Do case plans reflect the results of risk assessments? Are the intervention plans implemented in ways that are consistent with the original case plans/risk assessments? A non‐exhaustive list of potential outcomes might include those captured by process evaluations and other types of study exploring how closely the implementation of tools or approaches mirror their underlying logic. This might include examinations of whether practitioners working within RNR approaches are sensitive to all three elements of the RNR approach (Viljoen et al., 2018), and whether specific tools and approaches enable case managers to more effectively and/or efficiently match intervention components to risks and needs (College of Policing, 2021b); evidence of how specific tools, methods and procedures are used in practice (e.g., Salman & Gill, 2020), such as how different tools are used together; and levels of compliance with case management tools in practice (Schaefer & Williamson, 2018). Data relevant to the various factors that contribute to how tools and approaches are being implemented (i.e., Question 2b) will be drawn from studies discussing those implementation factors and moderator factors outlined in Section 1.2.6 above. It might also include outcome data from studies capturing practitioner or client feedback relating to, for example, whether tools or approaches require any adaptation (e.g., Salman & Gill, 2020).

#### 3.1.2 Types of study design

##### Evaluating effectiveness at countering radicalisation to violence (Question 1)

Only quantitative studies using experimental (e.g., randomised controlled trials) or ‘stronger’ quasi‐experimental research designs (as listed in guidance such as the UK government's *Magenta Book* (HM Treasury, 2020) will be eligible for inclusion in the analysis of question 1. Following previous Campbell systematic reviews relating to counter‐radicalisation (e.g., Mazerolle et al., 2021) and the *Magenta Book*, eligible quasi‐experimental designs include:

– Cross‐over designs;
– Regression discontinuity designs;
– Designs using multivariate controls (e.g., multiple regression);
– Matched control group designs;
– Unmatched control group designs where the control group has face validity;
– Unmatched control group designs allowing for difference‐in‐difference analysis; and
– Time‐series designs.

Whilst these quasi‐experimental designs are considered less robust than experimental research designs, they are increasingly being cited as potential solutions to long‐standing and well‐documented evaluation challenges in the field of counter‐radicalisation (Braddock, 2020). For both quasi‐experimental and experimental designs, eligible comparator/control group conditions will include treatment‐as‐usual; no treatment; and alternative treatment.

Data drawn from studies using experimental designs will be analysed separately to data drawn from studies using quasi‐experimental designs. Qualitative research will not be included.

##### Evaluating implementation (Question 2)

Both quantitative and qualitative research will be included when analysing how case management tools and approaches are being implemented in practice (Q2a); and those factors that influence whether tools and approaches are implemented as‐intended/expected (Q2b).

###### Quantitative research

Where relevant, experimental and those stronger quasi‐experimental designs outlined above will be included in the analysis of Question 2. However, as this question is focused on assessing the process of implementation, and not on evaluating impact, quantitative studies with weaker quasi‐experimental or non‐experimental designs will also be included in this analysis.

Only evidence relating to implementation from quantitative studies with ‘stronger’ research designs (e.g., experimental or stronger quasi‐experimental) designs will be included in the meta‐analysis for question 2. Quantitative studies with weaker studies will only be included in the qualitative analysis as outlined in the data analysis section later in the protocol.

###### Qualitative research

Qualitative and mixed‐methods research will be included in our analysis for question 2, with qualitative and quantitative data analysed separately. Qualitative data is crucial for understanding how practitioners are using tools such as the ERG 22+ to monitor client progress and outcomes (Salman & Gill, 2020); for capturing how case management interventions are delivered on the ground (e.g., Cherney & Belton, 2021a); and, as argued by Higginson et al. in their systematic review of gang‐related interventions, for capturing ‘the broadest range of evidence that assesses the reasons for implementation success or failure’ (Higginson et al., 2015, p. 22). Qualitative data is also needed to capture sources of additional data (including qualitative assessments) that case managers may use to supplement formalised measurement tools when assessing client progress (Lloyd, 2019). Studies drawing on qualitative interviews will also be crucial for capturing implementation data such as practitioner feedback on different tools and approaches (e.g., Chantraine & Scheer, 2021).

#### 3.1.3 Treatment of qualitative research

As discussed above qualitative research will be included when assessing processes of implementation (i.e., Research Questions 2a and 2b). However, qualitative data will be examined separately to quantitative data (see Data Analysis section) and will not be included in any meta‐analysis (i.e., qualitative data will not be converted into quantitative metrics).

#### 3.1.4 Publication types

Both peer‐reviewed and non‐peer‐reviewed/grey literature studies will be included.

#### 3.1.5 Types of intervention

Part I of the review will examine studies that report on *case management interventions, and their associated tools and approaches, seeking to counter radicalisation to violence*. This will include ‘standalone’ case management interventions, as well as programmes in which case management is one of many components being used to tackle radicalisation to violence.

To be included in Part I of the review, studies must report on primary research which concentrates on case management interventions and/or components of case management interventions (e.g., risk assessment, case planning etc.) with the aim of countering radicalisation to violence. Interventions must meet two inclusion criteria:

1. They must focus on individual clients (and not communities or collectives) and meet the definition of a case management intervention outlined above (i.e., incorporate the specific elements of case management listed above).^3^
2. They should be designed to counter radicalisation to violence. Interventions must seek to prevent at‐risk individuals from engaging in violent extremism and/or promote the disengagement of those who are already engaged in violent extremism (i.e., secondary and tertiary interventions). Where possible, this review will also examine evidence relating to how ‘at risk’ individuals are assessed as being eligible for an intervention, as well as the specific measures used to assess an individual's progress and outcomes. This review excludes primary interventions as they are focused on delivering client‐level outcomes whose relationship to violence is often less explicit (Marsden et al., 2017).

These inclusion criteria have been designed to ensure that Part I of the review is able to capture evidence from studies that report on case management interventions in their entirety, and those exploring the delivery and/or effects of individual components of the case management process. Examples of this type of research might include primary research studies that:

– *Examine the implementation or impact of violent extremism risk assessment tools*. Studies examining a) how practitioners use tools to assess clients, develop intervention plans or monitor and evaluate client progress and outcomes (e.g., Chantraine & Scheer, 2021; Salman & Gill, 2020) or b) the effects tools have on preventing negative outcomes (e.g., Viljoen et al., 2018) will be included. Studies examining the psychometric properties of tools will be excluded as that literature is the subject of a separate Campbell review (Hassan et al., 2022).
– *Examine the internal logic of the case management process*. Studies examining the relationship between different stages of the case management process, such as whether client assessments inform case planning or intervention delivery (e.g., Viljoen et al., 2019), will be included to understand how the process works in practice.
– *Compare the effectiveness of different tools/approaches based on how they are being delivered in practice*: Studies that, for example, compare the effectiveness of RNR, strengths‐based or desistance‐focused case management approaches (see Wong & Horan, 2021); different approaches to risk assessment (see Lloyd, 2019); or specific case management tools (e.g., Herzog‐Evans, 2018) will be included.

By including studies that report on individual components of case management, this review will be able to map the strength of the evidence base across the different stages of the case management process as presented in Figure 1, and identify key areas for future research.

#### 3.1.6 Duration of follow‐up

There will be no restrictions on the length of follow up when considering study eligibility for either research question in Part I. Where there are differences in the follow‐up period used across eligible studies, studies using comparable follow‐up periods will be synthesised, with studies using different follow up periods (e.g., short, medium, long‐term) analysed separately.

#### 3.1.7 Types of settings

We will include research on *secondary interventions* working with those at risk of engagement in violent extremism, and *tertiary interventions* working to prevent further engagement in violent extremism and/or promote desistance and disengagement amongst those already involved in violent extremism. There are no exclusion criteria on the context/setting in which an intervention is delivered, and a variety of populations will be included in the review. This will range from interventions working with individuals in community settings who have not yet committed an offence, and those working with individuals in post‐conviction contexts.

#### 3.1.8 Population

There are no demographic exclusion criteria.

Empirical studies engaging with practitioners, stakeholders and clients will be included in the review, but case management interventions working with victims of violence will be excluded. The full text coding scheme used by the reviewers will identify the population of the study (i.e., clients; stakeholders; practitioners; or some combination of different populations) in order to separate out key findings and conclusions relevant to each population. Where possible, other contextual variables (e.g., the type of practitioner; the delivery context etc.) will also be coded.

#### 3.1.9 Countries

There are no geographical exclusion criteria.

#### 3.1.10 Languages

Research in languages other than English will be reviewed, including studies in Russian, French, Danish, Norwegian, Swedish and German.

#### 3.1.11 Date of publication

Only studies published after January 2000 will be included on the basis that the term ‘radicalisation’ did not emerge in policy circles until the 21st Century (Neumann, 2013).

### 3.2 Search strategy (English language literature)

Relevant literature will be identified using a five‐stage search strategy that has been developed to identify relevant academic and grey literature:

1. *Targeted keyword searches* of academic databases (see Table 4).
2. *Hand searches* of key journals (see Table 5), research outputs of relevant research institutions/professional agencies (Table 6), and clinical trial repositories (Table 7).
3. *A review of studies cited in key evidence synthesis papers* (see Table 8).
4. *Consulting members of the research team and advisory board* to identify studies.
5. *Forward and backward citation searching* of studies identified at Stages 1–4.

**Table 4:** Platforms/providers included in review.

Platform/provider	Specific databases searched (if applicable)	Search fields^a^	Resource type
Ovid	PsycInfo	ab,hw,id,mh,ot,ti.	Principal
Elsevier	Scopus	TITLE‐ABS‐KEY	Principal
Web of Science	Book Citation Index (Social Sciences & Humanities).	Topic	Principal
	Social Sciences Citation Index.		
	Arts & Humanities Citation Index		
	Emerging Sources Citation Index.		
	Conference Proceedings Citation Index (Social Sciences & Humanities)		
	Medline		
EBSCO*host*	Criminal Justice Abstracts	Title	Principal
		Abstract	
		Keywords	
		Subject	
ProQuest	International Bibliography of the Social Sciences	ti, ab, mainsubject	Principal
ProQuest	Sociological Abstracts	ti, ab, if	Principal
Informit	CINCH: Australian Criminology Database	All Fields	Supplementary
ProQuest	Dissertations and Theses Global	ti, ab, mainsubject, diskw	Principal
EThOS (Dissertations)	N/A	All fields	Supplementary
Directory of Open Access Journals (DOAJ)	N/A	All fields	Supplementary

^a^ Whilst it might be preferable to search across all search fields in every database, it would be impractical to do so in the larger databases (e.g., Scopus, Web of Science) as the number of records returned would be too large to sift. These search fields are tailored to the size of each database.

**Table 5:** Key journals—Radicalisation & counter‐radicalisation.

Journal name
*Terrorism and Political Violence*
*Studies in Conflict & Terrorism*
*Behavioral Sciences of Terrorism and Political Aggression*
*Critical Studies on Terrorism*
*Journal for Deradicalization*
*Perspectives on Terrorism*
*International Journal of Conflict & Violence*
*Dynamics of Asymmetric Conflict*
*Journal of Policing, Intelligence & Counter Terrorism*
*Journal of Threat Assessment and Management*

**Table 6:** Grey literature^a^.

Source	Description
Institute for Strategic Dialogue (ISD) https://www.isdglobal.org/	Think‐tank
Hedayah https://www.hedayahcenter.org	Research centre
Royal United Services Institute (RUSI): https://rusi.org/	Think‐tank/Research centre
RAND https://www.rand.org/	Think‐tank
International Centre for Counter‐Terrorism (ICCT) https://icct.nl/	Research centre
Resolve Network https://www.resolvenet.org/	Research centre
Global Center on Cooperative Security https://www.globalcenter.org/	Research centre
International Centre for the Study of Radicalisation (ICSR): https://icsr.info/	Research centre
Centre for Research and Evidence on Security Threats (CREST) https://crestresearch.ac.uk	Research centre
National Consortium for the Study of Terrorism and Responses to Terrorism (START) https://www.start.umd.edu/	Research centre
IMPACT Europe http://impacteurope.eu/	Research repository
CT‐MORSE https://ct-morse.eu/	Research repository
National Criminal Justice Reference Service (NCJRS) https://www.ojp.gov	Research repository
Radicalisation Research https://www.radicalisationresearch.org	Research repository
VOX‐Pol https://www.voxpol.eu	Research repository
Crime Solutions https://crimesolutions.ojp.gov	Research repository
College of Policing Crime Reduction Toolkit https://www.college.police.uk/research/crime-reduction-toolkit	Research repository
Global Policing Database https://gpd.uq.edu.au/s/gpd/page/about	Research repository
Europol https://www.europol.europa.eu/	Government agency
Public Safety Canada https://www.publicsafety.gc.ca	Government agency
Department for International Development:	Government agency
Research for Development https://www.gov.uk/research-for-development-outputs	
Radicalisation Awareness Network https://ec.europa.eu/home-affairs/networks/radicalisation-awareness-network-ran_en	Government agency

^a^ To finalise this list, a long‐list of potential websites was first developed by the primary authors (JL and SM), and preliminary searches of each website conducted. Websites were excluded if the preliminary searches did not identify any potentially relevant primary research studies.

**Table 7:** Clinical trial registries^a^.

Source
Australian and New Zealand Clinical Trials Registry
ClinicalTrials.gov
Clinical Trials Results
Cochrane Central Register of Controlled Trials (CENTRAL)
ISRCTN Registry (controlled-trials.com)
NIH RePORTER
Trials Register of Promoting Health Interventions (TRoPHI)
Unreported Trials Register
UK Clinical Research Network (UKCRN Study Portfolio)
WHO International Clinical Trials Registry Platform

^a^ List of registries drawn from Fay and Eggins (2019). PROTOCOL: Family treatment drug courts for improving parental legal and psychosocial outcome. *Campbell Systematic Reviews*, 15, e1024.

**Table 8:** Key evidence synthesis studies: Counter‐radicalisation.

Source
Davies, M., Warnes, R. & Hofman, J. (2017). *Exploring the transferability and applicability of gang evaluation methodologies to counter‐violent radicalisation*. Cambridge: RAND Europe.
Feddes, A. & Gallucci, M. (2015). A literature review on methodology used in evaluating effects of preventive and de‐radicalisation interventions. *Journal for Deradicalization*, 5, 1–27.
Hassan, G., Brouillette‐Alarie, S., Ousman, S., Kilinc, D., Savard, É. L., Varela, W., Lavoie, L., Fetiu, A., Harris‐Hogan, S., Borokhovski, E., Pickup, D., Madriaza, P., Rousseau, C., Thompson, S. K., McCoy, J., Venkatesh, V., Boivin, M., Srimathi Narayana, M., Morin, D., & the CPN‐PREV team. (2021a). *A systematic review on the outcomes of primary and secondary prevention programs in the field of violent radicalization*. Canadian Practitioners Network for the Prevention of Radicalization and Extremist Violence.
Hassan, G., Brouillette‐Alarie, S., Ousman, S., Savard, É. L., Kilinc, D., Madriaza, P., Varela, W., Pickup, D., Danis, E., & the CPN‐PREV team. (2021b). *A systematic review on the outcomes of tertiary prevention programs in the field of violent radicalization*. Canadian Practitioners Network for the Prevention of Radicalization and Extremist Violence.
Lewis, J. & Marsden, S. (2021). *Countering Violent Extremism Interventions: Contemporary Research*. Lancaster University, Lancaster: Centre for Research and Evidence on Security Threats (CREST).
Lewis, J., Marsden, S. & Copeland, S. (2020). *Evaluating Programmes To Prevent And Counter Extremism*. Lancaster University, Lancaster: Centre for Research and Evidence on Security Threats (CREST).
Mastroe, C. & Szmania, S. (2016). *Surveying CVE Metrics in Prevention, Disengagement and Deradicalization Programs*. University of Maryland: START.
Pistone, I., Eriksson, E., Beckman, U., Mattson, C. & Sager, M. (2019). A scoping review of interventions for preventing and countering violent extremism: Current status and implications for future research. *Journal for Deradicalization*, 19, 1–84.
Morrison, J. F., Silke, A., Maiberg, H., Slay, C., & Stewart, R. (2021). *A Systematic Review Of Post‐2017 Research On Disengagement And Deradicalisation*, Lancaster University, Lancaster: Centre for Research and Evidence on Security Threats (CREST).
van Hemert, D., van den Berg, H., van Vliet, T., Roelofs, M., Huis in ‘t Veld, M., Marret, J., Gallucci, M. & Feddes, A. (2014). *Innovative Method and Procedure to Assess Counter‐violent‐radicalisation Techniques in Europe: Synthesis report on the state‐of‐the‐art in evaluating the effectiveness of counter‐violent extremism interventions*. IMPACT Europe Report.
Zeuthen, M. (2021). *Reintegration: Disengaging violent extremists: A systematic literature review of effectiveness of counter‐terrorism and preventing and countering violent extremism activities*. Report commissioned and financed by the Policy and Operations Evaluation Department (IOB) of the Netherlands Ministry of Foreign Affairs.

#### 3.2.1 Key word searches

In consultation with the broader research team, the primary authors (SM and JL) have developed a comprehensive list of key words that will be used to search the academic databases listed in Table 4. These databases have been categorised as either *principal* or *supplementary* resources. Following the approach taken by Gusenbauer and Haddaway's (2020) in their categorisation of 28 academic search systems for systematic reviews, the databases listed below are categorised according to the functionality and replicability of their search functions. Primary resources refer to those platforms/databases that provide a sophisticated search functionality which allows for searching different combinations of key words across multiple search fields in a way that can be replicated by other researchers. In contrast, secondary resources are those platforms/databases offering a more limited search functionality that does now allow for more comprehensive key word searches, or for the replication of searches.^4^

The full key words search strategy as outlined below will first be replicated across the principal databases listed in Table 4 (adapted to database‐specific parameters) before searches using more limited keyword combinations are conducted in supplementary databases. The same databases will be used across both parts of the review. Supplementary resources will be used ‘in addition to a principal resource for its specific qualities that could retrieve additional records and to further improve the evidence base’ (Gusenbauer & Haddaway, 2020, p. 196).

To understand the scale of the literature, a preliminary search was conducted on 20/05/21 in PsycNet using key words categorised under two search domains: ***Problem*** (Any Field: extremis* OR Any Field: terror* OR Any Field: radical*) and **Intervention** (Any Field: prevent* OR Any Field: treat* OR Any Field: interven*). Search results were sorted on relevance, and a skimming review of the abstracts and titles of the first 100 records was conducted. This exercise identified a large number of irrelevant studies that either a) did not discuss a case management tool or approach; or b) did not conduct empirical research. In response, a range of alternative search strategies were piloted across multiple academic databases (PsycNet; Scopus; Web of Science; Academic Search Complete) to increase the relevance of the results. A further stage of piloting was conducted between the 3rd and 5th May 2022 following feedback from the Campbell Crime and Justice editorial board. Based on this piloting process, the search strategy will include three domains as outlined below:

– *Problem*: Key words relevant to violent extremism and its synonyms (Radicali*, extremis*, terroris*); or specific ideologies (e.g., ‘far‐right’; ‘white supremacis*).
AND
– *Intervention*: Key words describing synonyms for *interventions* (e.g., ‘interven*’; ‘program*’ etc.) and *tools* (e.g., ‘tool’; ‘instrument’); and the different *stages* of the case management process (e.g., ‘refer*’; ‘assess*’);
  AND
– *Outcomes*: Key words relating to primary *outcomes* of prevention (e.g., ‘Prevent*’); and desistance (e.g., ‘disengage*’)

#### 3.2.2 Hand searches

A number of key journals, grey literature databases, and research repositories will be hand‐searched, with the specific approach taken to searching each source tailored to the functionality (i.e., the specific filters that can be used or search fields available) of its website.

#### 3.2.3 Review of existing synthesis studies

The bibliographies of the studies listed in Table 8 will also be reviewed. These studies were identified by the research team as the most comprehensive overviews of empirical research into interventions seeking to counter radicalisation to violence and related phenomena.

#### 3.2.4 Drawing on the expertise of the review team and review advisory board

The review team and the review's advisory board has a strong knowledge of existing empirical research into interventions seeking to counter radicalisation to violence operating in a number of different continents. One member of the review team (A. C.) also has specific expertise on the use of case management tools and approaches for countering radicalisation to violence (e.g., Cherney & Belton, 2020, 2021a, 2021b; Cherney, 2021). Once the stages of literature searching outlined above have been completed, the review team will identify additional studies known to them not yet identified. Members of the advisory board will also be asked to do the same.

#### 3.2.5 Forward and backward citation searching

Once the studies identified through the first four search strategies described in Sections 3.2.1–3.2.4 above have been screened (see Section 3.4 below), forward and backward citation searches will be conducted on all studies deemed to be eligible for inclusion in the review.

Forward citation searches will be conducted in Google Scholar. Backward citation searches will be conducted by manually searching the reference lists of included studies. Additional studies identified through these searches will be screened, and eligible studies included in the review. Forward and backward citation searches will then be undertaken on these additional studies. This process will repeat until forward and backward citation searches are exhausted (i.e., when searches no longer return any additional studies that meet our inclusion criteria).

### 3.3 Search strategy (non‐English language literature)

The research team includes researchers who are fluent in those languages listed in Section 3.1.8, and who will be responsible for searching for research in languages other than English (LOE). As there are no concrete guidelines for how LOE literature searches should be conducted when undertaking a systematic review (Walpole, 2019), each language specialist will use a search strategy which has been adapted from the methodology presented above. However, the LOE search strategy will not be as comprehensive given that identifying, translating and analysing LOE literature will be more resource intensive (Walpole, 2019).

(1) Team members will translate the English key words into the specific languages assigned to them, and will identify alternative terms for any key words for which a direct translation does not exist. These language‐specific key words will be used to search for literature in academic databases containing research in that language.
(2) This will include searching language‐specific databases (see Walpole, 2019), or databases such as Scopus that include research in multiple languages. Each language specialist will be responsible for identifying databases relevant to them, and a complete list of all databases searched (and search results) will be included in the final review.
(3) Where relevant, language specialists will also hand search key journals published in LOE (such as EXIT‐Journal Deutschland) and LOE research outputs of relevant research institutions and professional agencies known to them. Again, each team member will be responsible for identifying and recording relevant sources of this type.
(4) Each specialist will be asked to identify other studies known to them. As recommended by Walpole (2019), we will also ask members of the advisory board for more informal support in identifying potentially relevant studies that have yet to be identified.

### 3.4 Description of screening process

#### 3.4.1 English language literature

English language studies will be screened using a five‐stage process:

1. All studies identified through the search strategy above will be imported into EndNote. Duplicate records will be removed before the initial list is uploaded into Covidence.
2. One reviewer (JL) will use Covidence to review all titles and exclude duplicate and obviously irrelevant studies (i.e., those that are completely unrelated to radicalisation).
3. Two reviewers (JL and ML) will then use Covidence to review all remaining abstracts to exclude obviously irrelevant studies (i.e., those unrelated to radicalisation). Both reviewers will then conduct a second stage of abstract screening using a screening tool (see Appendix II). An online meeting will be arranged to resolve any conflicts. A third reviewer (SM) will make the final decision in the event that conflicts are not resolved.
4. The same two trained reviewers (JL and ML) will conduct a full text screening of all remaining studies in Covidence, guided by the same screening tool. Once the full‐text screening has been completed, the two reviewers will meet (either in person or online) to review their decisions, and to resolve any conflicts. In the event that a conflict cannot be resolved, a senior reviewer (SM) will make the final decision. SM will also review the final list of included studies against the inclusion criteria above.

#### 3.4.2 Non‐English language literature

Non‐English language studies will be screened using a four‐stage process. The same screening and data extraction tools will be used for both English and non‐English language studies:

1. The titles of all studies in languages other than English will be reviewed by the primary LOE reviewer with the relevant language skills, and clearly irrelevant studies excluded (i.e., those entirely unrelated to radicalisation to violence).
2. The abstracts of remaining studies will then be screened by the same primary LOE reviewer, and by a secondary LOE reviewer with relevant language skills. If necessary, an online meeting will be arranged to resolve any conflicts. SM will again make the final decision on any outstanding conflicts.
3. The above process will then be repeated for full‐texts.
4. Once the screening process has been completed, the primary LOE reviewer will translate the abstract and full‐texts of each remaining non‐English language study into English. The primary English‐language reviewer (JL) will then check the full‐text and abstract of each study against the inclusion criteria, and, if necessary, an online meeting held with the primary LOE reviewer to discuss any studies that do not appear to meet the inclusion criteria. SM will again make the final decision on conflicts.

### 3.5 Study coding

A data extraction and coding tool has been developed to guide the full‐text coding process (see Appendix III). This tool will capture key details about each individual study; descriptive information about the specific interventions, tools and/or approaches examined by each study; and relevant information relating to their effectiveness and implementation. English language studies will be double‐blind coded by the primary reviewers (JL and ML) during the full‐text screening stage. LOE studies will be double‐blind coded by one of the primary reviewers (JL, using a translation) and the primary LOE language specialist. An online meeting will be arranged to make a final decision on any coding conflicts and, at this stage, a third reviewer (SM) will make the final decision on any remaining conflicts.

### 3.6 Assessing risk of bias

#### 3.6.1 Studies included in analysis related to Research Question 1

Included studies will be assessed using either Version 2 of the Cochrane risk‐of‐bias tool for randomized trials (RoB 2) or the Cochrane Risk Of Bias In Non‐Randomized Studies—of Interventions (ROBINS‐I) tool (Sterne et al., 2016, 2019). Both tools incorporate a range of ‘signalling questions’ that are used to assess the risk of bias across relevant domains (see below), and in turn to inform an overall risk of bias assessment for the results of each study.

##### Randomised studies (RoB 2)

The RoB 2 tool is structured around five domains: 1) Bias arising from the randomisation process; 2) Bias due to deviations from intended interventions; 3) Bias due to missing outcome data; 4) Bias in measurement of the outcome; and 5) Bias in selection of the reported result (Sterne et al., 2019). Studies will be assessed against each domain, with each domain awarded an overall risk of bias judgement (high/low/some concerns). Each study will then be awarded an overall risk of bias as outlined in Table 9 below. Following Mazerolle et al. (2020) the results of these assessments will be presented using summary tables and a risk of bias summary figure and, if possible, sensitivity analysis will be conducted to understand the impact of RoB results. The way in which these assessments are incorporated into the statistical analysis as outlined in Section 3.8 below will be determined by a) the number of studies identified; and b) the level of variation in risk of bias identified across the included studies. Following Mazerolle et al. (2020), this analysis ‘may be stratified by level of risk or all studies may be included in one analysis with a narrative discussion of the risk of bias’ (p. 8).

**Table 9:** Risk of bias assessment results (Sterne et al., 2019).

Assigned risk level	Description
Low risk of bias	Study assessed as having a low risk of bias for all domains
Some concerns	Study raises some concerns in relation to at least one domain, but is not assessed as having a high risk of bias for any domain.
High risk of bias	Study assessed as having a high risk of bias for at least one domain; or concerns raised across multiple domains serve to lower confidence in the overall result.

##### Non‐Randomised studies (ROBINS‐I)

The ROBINS‐I tool is structured around seven domains that capture the risk of bias pre‐intervention; at‐intervention; and post‐intervention: **Pre‐Intervention**: (1) Bias due to confounding; (2) Bias in selection of participants into the study; **At‐Intervention**: (3) Bias in classification of interventions; **Post‐Intervention**: (4) Bias due to deviations from intended interventions; (5) Bias due to missing data; (6) Bias in measurement of outcomes; (7) Bias in selection of the reported result (Sterne et al., 2016). Studies will be assessed against each domain, with each domain awarded an overall risk of bias judgement (low/moderate/serious/critical/no information). Each study will then be awarded an overall risk of bias as outlined in Table 10.

**Table 10:** Risk of bias assessment results (Sterne et al., 2016).

Assigned risk level	Description
Low risk of bias	Study assessed as having a low risk for all domains
Moderate risk of bias	Study assessed as having low or moderate risk for all domains
Serious risk of bias	Study assessed as having serious risk of bias in at least one domain, but not at critical risk of bias in any domain.
Critical risk of bias	Study assessed having critical risk of bias in at least one domain

The methods used to summarise the results of risk of bias assessments and to incorporate risk of bias into the statistical analysis will follow the approach outlined in the discussion of RoB 2 above. Evidence from randomised and non‐randomised studies will be analysed separately.

#### 3.6.2 Studies included in analysis related to research question 2

Studies using experimental or the stronger quasi‐experimental designs as outlined in Section 3.1.2 will be assessed using either the ROBINS‐I or RoB 2 tool, with the specific tool used dependent on the research design of the individual study. Weaker quantitative studies will be assessed using the Effective Public Health Practice Project (EPHPP) Quality Assessment Tool for Quantitative Studies.^5^ This tool is organised a series of questions that are used to assess the quality of eligible studies across domains related to selection bias; study design; confounders; blinding; data collection methods; and withdrawals and dropouts.

Following Mazerolle et al. (2020), eligible qualitative studies will be assessed using a checklist developed by the Critical Appraisal Skills Programme (CASP).^6^ This checklist is organised around ten specific questions that guide decision‐making about the quality of qualitative research studies (see Appendix II). In line with Mazerolle et al. (2020), studies for which either of the below question is ‘No’ or ‘Can't Tell’ will not be included in the evidence synthesis:

– Is the research design appropriate to answer the question?
– Was the sampling strategy appropriate to the aims of the research?

### 3.7 Criteria for determination of independent findings

The analysis of data relevant to both research question 1 and 2 will use a similar approach for mitigating three issues of dependence as identified by Mazerolle et al. (2020): multiple documents reporting on a single empirical study; conceptually similar outcomes being reported in the same document; and studies including clustering in their research design.

Where multiple studies reporting on the same empirical findings (i.e., are drawn from the same research project) meet the inclusion criteria for this review, each study will be coded separately, but only the study presenting the most complete data will be included. Documents will be assessed as reporting on the same empirical findings based on examining the extracted data for any identifiable overlap in document authors; the intervention design; the research conditions; and any references to other eligible studies identified through the searches.

Studies identified for inclusion in a systematic review often report different measures for outcomes that are conceptually similar. We will transform the smallest number of effect sizes to a standardised effect size in compliance with Campbell guidelines (Polanin & Snilstveit, 2016). This way the final effect size metric will be that which is calculated most commonly for each outcome. Finally, if studies use clustered research designs, we follow Mazerolle et al. (2020) in using Higgins et al. (2011) to adjust the standard error for the purposes of analysis.

### 3.8 Statistical procedures and conventions/data synthesis

Evidence relating to research questions 1 and 2 will be analysed and presented separately. The data synthesis procedure for both questions will include the following methods:

**Table:** 

Research Question 1	Research Question 2
Narrative summary of evidence	Narrative summary of evidence
Meta‐analysis of data from experimental and stronger quasi‐experimental studies	Meta‐analysis of data from experimental and stronger quasi‐experimental studies
Alternative method of quantitative synthesis using methods approved by Cochrane (see Section 3.8.3) of outcome data that cannot be included in the meta‐analysis.	Alternative method of quantitative synthesis using methods approved by Cochrane (see Section 3.8.3) of outcome data that cannot be included in the meta‐analysis.
	Framework synthesis of qualitative data

#### 3.8.1 Narrative summary of the evidence base (Research question 1 and 2)

The first stage of data analysis will consist of qualitatively mapping the different case management tools and approaches identified in the research, and the different progress and outcome measures used to (a) assess the effectiveness of each tool/approach in countering radicalisation to violence (research question 1); and (b) processes of implementation (research question 2). Evidence relating to the different research questions will be analysed and summarised separately. The analysis of both questions will consist of four stages:

1. The different case management tools and approaches examined across the included studies will be identified, and a typology of different tools and approaches will be created. This typology will initially be developed by JL, and reviewed by SM.
2. Narrative summaries describing how each tool/approach is intended to work will be developed. These summaries will be informed by the template for intervention description and replication (TIDieR) (Hoffmann et al., 2014). If possible, this will include mapping tools against different approaches and different stages of the case management process as outlined above.
3. Primary and secondary outcome measures relevant to each research question will be identified, and a typology of different measures created.
4. Outcome measures will be mapped against the different tools and approaches.

#### 3.8.2 Meta‐analysis (Research question 1 and 2)

Statistical analysis may not be possible given the known weaknesses in the evidence base underpinning current efforts to evaluate counter‐radicalisation interventions. However, where possible, we will conduct meta‐analyses of relevant outcome measures relating to research question 1 (countering radicalisation to violence) and 2 (implementation). As evidence relating to research question 1 will be drawn solely from experimental and stronger quasi‐experimental studies, all relevant studies will be eligible for inclusion in the meta‐analysis, provided the reported outcome data meets the criteria outlined below. However, whilst the qualitative analysis of research question 2 will include studies using weaker quasi‐experimental and non‐experimental designs, the meta‐analysis of implementation outcomes will only include data from experimental or stronger quasi‐experimental designs. An example of such a study would be an experimental or quasi‐experimental study comparing how different groups of practitioners/practitioners working in different contexts implemented a specific tool or approach. As noted above, we will only include studies building on independent findings in the meta‐analysis. Therefore, studies reporting on the same empirical findings, or conceptually similar outcomes in the same study will only be included once.

We expect that this review will need to consider multiple factors (particularly different case management tools and approaches) and various outcomes in order to capture the likely variety in how outcomes related to disengagement, desistance or deradicalisation (research question 1), and processes of implementation (research question 2) are captured by different studies. Given the broad spectrum of case management tools and approaches that are likely being used to counter radicalisation to violence and the large amount of potential outcome variables relating to each of the research questions we will run meta‐analyses for all factors and outcome combinations for which at least two inputs from independent samples can be identified. In general, we follow protocols of Campbell systematic reviews on related topics as guides (e.g., Mazerolle et al., 2021; Wolfowicz et al., 2021).

In cases where there is no focus on just one factor or outcome, it is general practice to use bivariate correlations for imputing effect size. Using bivariate correlations to compute effect sizes has advantages when compared with effect sizes taken from multivariate statistical models as they are more consistent and less contaminated (Pratt et al., 2014). Whenever the identified studies do not provide sufficient information (descriptive data or correlation matrices) we will contact the authors of these studies in an effort to receive the information needed. If we cannot obtain this data from the authors we will exclude the study from the quantitative meta‐analysis. However, the study will be included in the qualitative assessment. In cases where a correlation matrix is not available but descriptive statistics are sufficiently detailed, we will calculate the effect sizes using the appropriate formulas for that type of data. In doing so we will follow Lipsey and Wilson (2001) and use the ‘Practical Meta‐Analysis Effect Size Calculator’ that is accessible on the Campbell Collaboration website.^7^

If research projects produce results on follow‐up periods, separate meta‐analyses will be conducted for each follow‐up period for which data is available, and outcome data drawn from studies using comparable follow‐up periods will be synthesised. Where a single study presents data for multiple follow‐up periods, effect sizes from each time point will be analysed separately and synthesised with studies using comparable follow‐up periods.

Since it is an inclusion criterion that the studies focus on individuals, it is reasonable to expect that evaluations will usually report outcomes as a continuous measure (e.g., self‐reported measures on a Likert scale). In these instances, we will calculate Hedges' g (standardised mean differences) directly. However, should evaluations report binary outcomes (e.g., whether or not participants disengage), we will compute effect sizes as odds ratios. The odd ratios will be then transformed into Hedges’ g for the meta‐analyses (see Borenstein et al., 2009).

As recommended by the Cochrane handbook (Deeks et al., 2022), we will use the metafor package for R (Viechtbauer, 2010) or Stata (StataCorp, 2021) to conduct random effects inverse variance meta‐analyses (Lipsey & Wilson, 2001). In line with previous Campbell Reviews we will report mean effect sizes with confidence intervals throughout the text and add forest plots for each meta‐analysis. For each meta‐analysis, we will also check for heterogeneity with the heterogeneity measures Q (the weighted sum of squares about the fixed effect estimate) and I^2^ (Higgins & Thompson, 2002). We will then do subgroup analyses to investigate the origin of the heterogeneity. If possible, we will run moderator analyses to compare effect sizes produced by studies using different research designs. Where the data allows, we will also run moderator analysis on different categories of moderator and implementation factor as discussed in Section 1.2 above. However, we anticipate that the likely small number of studies identified will prohibit the use of any type of moderator analysis.

A key limitation of this analysis relates to the extent to which observed outcomes can be attributed to the use of a specific case management tool. Whilst case management *approaches* aim to deliver primary or secondary outcomes that are directly relevant to countering radicalisation to violence, case management *tools* serve a different purpose. Case management tools are not designed to counter radicalisation to violence but to support case management interventions in doing so. For example, a risk assessment tool does not directly prevent an individual from becoming engaged in violent extremism, but can support case managers in designing intervention plans that are specifically designed for this purpose. As a result, it may not be possible to say that a specific case management tool is effective at countering radicalisation to violence. However, whilst it may not be possible to isolate the specific contribution of a case management tool in delivering an observed outcome, it may be possible, depending on the evidence identified, to explore whether interventions that a) use specific case management tools; and b) do so in a way that is consistent with their intended function are effective at delivering positive intervention outcomes (Viljoen et al., 2018).

#### 3.8.3 Synthesis of other quantitative data (Research question 1 and 2)

When outcome data drawn from experimental and stronger quasi‐experimental designs cannot be included in a meta‐analysis (i.e., when the data presented in the primary studies is incomplete), where possible, this data will be synthesised using one of the alternative methods to meta‐analysis that the Cochrane collaboration deems to be acceptable (McKenzie & Brennan, 2022). These methods include summarising effect estimates; combining P values; and vote counting based on the direction of effect. The specific method used will be determined by the data relevant to each effect size that is available. To ensure that synthesis is transparent and replicable, any alternative syntheses will be reported in ways that are consistent with the synthesis without meta‐analysis (SWiM) reporting guidelines (Campbell et al., 2020).

Where possible, the above approach will also be applied to data drawn from weaker quantitative studies included in the analysis of research question 2. However, weaker and stronger quantitative designs will be synthesised separately.

#### 3.8.4 Synthesis of qualitative evidence (Research question 2)

Qualitative evidence—as well as quantitative data that cannot be included in either form of quantitative synthesis outlined in 3.8.2 and 3.8.3 above—will be synthesised using the framework synthesis (Booth & Carroll, 2015) approach. This approach, as described by Mazerolle et al. (2020), uses ‘systematic rules or a framework to arrange data into distinct categories that are then synthesised using a variety of techniques such as tables, matrices, and narrative textual summaries’ (p. 11). This arrangement of data involves categorising data according to a pre‐existing framework (i.e., deductive coding), before conducting thematic analysis to synthesise any evidence that does not fit into this initial framework (i.e., inductive coding) (Pollock et al., 2020). Following this approach, data will first be coded using the coding framework outlined in Appendix III, before thematic analysis is conducted to synthesise evidence relating to the different case management tools or approaches identified.

### 3.9 Assessing transferability

Given the diversity within the field of countering radicalisation to violence as discussed earlier, this review will include evidence from interventions that are delivered in diverse contexts. The authors will therefore specifically assess whether the findings from Part I of this review are transferable to counter‐radicalisation interventions operating in different contexts. This question of transferability—or ‘whether the level of effectiveness (or perceptions and experiences) of an intervention in a specific setting or population will be similar to the observed level of effectiveness (or perceptions and experiences) observed in a systematic review’ (Munthe‐Kaas et al., 2019, p. 2)—is pertinent for two reasons.

First, there are a range of transfer factors (Munthe‐Kaas et al., 2020) that might influence how case management counter‐radicalisation interventions are delivered and the results they produce. This includes the national or local context, including the specific political, legal or socio‐economic system (Graham & Robertson, 2022), and the specific type of setting in which an intervention is delivered (Munthe‐Kaas et al., 2019). Most notably, there is a clear distinction between secondary and tertiary interventions in terms of the populations they work with, and the settings in which they are delivered (Elshimi, 2020; Marsden et al., 2017). The design of both types of intervention will also be influenced by the national, local and institutional contexts in which they are developed, and similar contextual factors will also shape how interventions are enacted on‐the‐ground (Harris‐Hogan, 2020; Lewis, 2020).

Second, there is no single model of case management, nor a single profile of a case management client, which means that evidence of effectiveness (or lack thereof) gathered from a subset of cases supported through one intervention does not necessarily mean that specific intervention will be effective in other contexts, or deliver better outcomes in case management interventions more broadly (Orwin et al., 1994). It will therefore be important to consider how specific contextual factors might have contributed to the success or failure of an intervention as‐a‐whole, and to the client‐level outcomes recorded in each individual case.

There is no standard approach for assessing the transferability of systematic reviews. However, in their mapping exercise of checklists for assessing the transferability of findings from primary or secondary research—including systematic reviews—Munthe‐Kaas, Nøkleby and Nguyen identified seven common themes: population, intervention, implementation context, comparison condition, outcomes, environmental context and researcher conduct (Munthe‐Kaas et al., 2019, p. 7). The transferability of the evidence included in this review will be qualitatively assessed against these themes as shown in Table 11 below. This table presents a non‐exhaustive list of factors that might be considered for each theme.

**Table 11:** Transferability themes (from Munthe‐Kaas et al., 2019).

Theme	Sub‐themes and description
Population	Population of interest
	At‐risk of engagement or already engaged in violent extremism
	Population characteristics
	Information about population‐of‐interest, such as their demographic characteristics; type of extremism; whether they have engaged with the intervention voluntarily etc.
Intervention	Intervention characteristics
	Information about intervention design, stage(s) of case management included, specific tools or approaches used etc.
	Intervention delivery
	Information on how intervention is intended to be delivered, such as the settings in which it is delivered, and whether the intervention can be tailored to other types of setting; intensity or duration of the intervention etc.
Implementation context	Providers
	Number and type of providers delivering an intervention.
	Organisations
	Information about implementing organisation(s) such as the resources available, size and structure; culture etc.
Comparison intervention (*if relevant*)	Information about the comparison condition against which an intervention is evaluated, including an assessment of whether the support provided through a control condition is of sufficient quality to provide a robust comparison of effectiveness.
Outcomes	Information about the specific outcomes an intervention is seeking to deliver, and how they are being measured.
Environmental context	Relevant information about, for example the *temporal context (*e.g., whether there have been any relevant changes in how an intervention operates/or the broader context since a study was conducted); the *political, social or regulatory* context; or other *interventions* that might influence the intervention in question.
Researcher conduct	Relevant information about how the research was conducted/how data was analysed which might influence results.

### 3.10 Assessment of publication bias of studies included in meta‐analysis

Following Mazerolle et al. (2020), publication bias will be assessed both visually and, if possible, statistically. Funnel plots will be used to assess asymmetry and, and any identified asymmetry examined through sub‐group analysis of published and non‐published studies.

If possible, statistical analysis of these sub‐groups will be conducted.

## 4 METHODOLOGY—PART II

The inclusion criteria for Part II have been informed by the criteria for Part I so as to maximise the synergies between them. However, there are important differences in the types of interventions and study designs that will be included, and the methods of data synthesis used. This section therefore describes the methodology used for Part II separately.

### 4.1 Criteria for including and excluding reviews

#### 4.1.1 Outcomes

Question 3 will examine outcomes relating to countering violence; and Question 4 will examine outcomes relating implementation. The outcomes relevant to these questions will be comparable to those used to assess effectiveness (Q1) and implementation (Q2) in Part I:

– *Outcomes relevant to countering violence (Q3)*: *Primary outcomes* will include measures of disengagement and desistance from violence captured using similar tools as outlined in Part I, including risk assessment tools; arrest or prosecutions data; case notes; and qualitative or quantitative data relating to recidivism. *Secondary outcomes* will also be comparable to Part I, and might include measures included in tools used to assess the risk of violence, such as the Historical, Clinical, and Risk Management‐20 (HCR‐20) scale (Douglas & Reeves, 2010).
– *Outcomes relevant to implementation (Q4)*: In line with Part I, implementation will be evaluated by reviewing evidence related to the different elements of implementation as outlined in Section 3.1.1 above.

#### 4.1.2 Types of study design

The main point of departure between Parts I and II will be the study designs included, as Part II will only include systematic reviews and meta‐analyses. The methodology for Part II has been informed by the Cochrane methodological guidelines for conducting an ‘overview of reviews’ (Pollock et al., 2021). These guidelines state that an overview of reviews is appropriate when authors want to ‘address research questions that are broader in scope than those examined in individual systematic reviews’; and when this approach is ‘associated with time and resource savings, since the component systematic reviews have already been conducted’. In line with these guidelines, an overview of reviews is appropriate for Part II on the basis that a) the analysis of transferability outlined above is outside the scope of a standard systematic review; and b) it would be impractical to review all primary research reporting on case management in the field of violence prevention within the timescale of this review.

In line with the Campbell collaboration definition of a systematic review,^8^ identified reviews must meet the below criteria to be eligible for inclusion in Part II of this review:

– Authors must specify *clear inclusion and exclusion criteria*, and offer justification.
– Authors must use an *explicit search strategy*, and specify:
  ○ The different strategies used to identify research (e.g., keyword searches of academic databases; citation searching; reviewing grey literature, etc.).
  ○ The specific sources used to identify literature.
  ○ The process of screening studies.
  ○ The number of records identified through the initial searches.
  ○ The number of unique records included in the review.
– Authors must have conducted a *systematic coding and analysis of included studies*.
  ○ The methods of coding and analysis should be clearly outlined, and justified.
  ○ Where relevant, methods used to conduct meta‐analysis must be specified.

To capture the most comprehensive range of evidence, systematic reviews including randomised and nonrandomised research designs will be included in Part II.

#### 4.1.3 Treatment of qualitative research

Qualitative research will not be included in Part II of the review.

#### 4.1.4 Publication type

Both peer‐reviewed and non‐peer‐reviewed/grey literature studies will be included in Part II.

#### 4.1.5 Types of interventions

To be included in Part II, reviews must report on *case management interventions and/or components of case management interventions* (e.g., risk assessment, case planning etc.) with the aim of *preventing engagement in, or promoting disengagement from collective or interpersonal violence*. Interventions must meet two inclusion criteria:

1. They must focus on individuals and meet the definition of a *case management intervention* as presented above, although the specific term used to describe the tailoring of interventions to individual cases may vary across different fields.
2. They must be specifically designed to *prevent violent offending or reoffending*. Interventions seeking to tackle both collective and interpersonal violence will be included:
  a. Collective Violence: Physical, psychological or sexual violence perpetrated by those acting as part of a collective such as gang‐related violence (e.g., Randhawa‐Horne et al., 2019) or larger‐scale militancy (e.g., USAID, 2021).
  b. Interpersonal Violence: Physical, psychological or sexual violence perpetrated by individuals (or small groups of individuals) against other people (Mercy et al., 2017), including family members or partners (e.g., Gondolf, 2008).

Reviews reporting on both standalone case management interventions as well as larger‐scale programmes in which case management is part of a broader suite of interventions will be included. An example of the latter might include ‘focused deterrence’ approaches in which the police and their partners simultaneously use diverse approaches such as preventive policing; case‐management; and communication campaigns to engage at‐risk youth or youth already engaged in gangs (e.g., Brantingham et al., 2021; Engel et al., 2013).

#### 4.1.6 Types of settings

In line with Part I, we will include reviews of *secondary interventions* working with those at risk of engagement in violence, and *tertiary interventions* working to tackle ongoing engagement in violent activity and/or promote desistance and disengagement amongst those who have already committed violence. There are no exclusion criteria on the context/setting in which an intervention is delivered, and a variety of populations will again be included.

#### 4.1.7 Population

There are no demographic exclusion criteria.

Reviews of studies engaging with practitioners, stakeholders and clients will be included in Part II, but case management interventions working with victims of violence will again be excluded. The full text coding scheme used by the reviewers will use the same criteria as in Part I to capture the population of the review as well as other contextual variables.

#### 4.1.8 Countries

There are no geographical exclusion criteria, and the languages included will mirror Part I.

#### 4.1.9 Date of publication

Only reviews published after January 2000 will be included to align with Part I of the review.

### 4.2 Search strategy (English language literature)

Relevant reviews will be identified using a four‐stage search strategy that has been developed to identify relevant published and non‐published academic and grey literature:

1. Targeted keyword searches of academic databases.
2. Hand searches of key journals (see Table 12).
3. Consulting members of the research team and advisory board to identify studies.
4. Forward and backward citation searching of studies identified at Stages 1‐3.

**Table 12:** Key journals—Violence prevention^a^.

Journal name
*Trauma Violence & Abuse*
*Annual Review of Criminology*
*Criminology*
*Journal of Interpersonal Violence*
*Youth Violence and Juvenile Justice*
*Justice Quarterly*
*Crime and Justice—A Review of Research*
*Aggression and Violent Behavior*
*Criminology & Public Policy*
*Journal of Quantitative Criminology*

^a^ These are the ten journals with the highest impact factor in category of ‘criminology and penology’ as listed in the Web of Science Journal Citation Report 2021.

#### 4.2.1 Key word searches

The key word search strategy for identifying literature for Part II of the review will be adapted from the Part I key word search strategy outlined above in the following ways:

– The *Problem* domain will be updated to capture key terms related to collective and interpersonal forms of violence.
– The *Intervention* domain will remain unchanged.
– The *Outcome* domain will be changed to include terms that are relevant to other forms of collective violence (e.g., ‘demobili*’).
– A *Data* domain will be added to ensure that the searches only capture studies referencing a review or meta‐analysis.

Key word searches will be conducted in the same databases used for Part I (see Table 4). In addition, the *Cochrane*, *Campbell* and *PROSPERO* databases will also be searched for relevant systematic reviews as they are the most comprehensive online databases of systematic reviews.

#### 4.2.2 Hand searches

A number of key journals will be hand‐searched, with the specific approach taken to searching each source tailored to the functionality (i.e., the specific filters that can be used or search fields available) on its website.

#### 4.2.3 Drawing on the knowledge of the advisory board

Several members of the advisory board for this review have a strong knowledge of research in the broader field of violence prevention. Once the initial stages of literature searching as outlined above have been completed, members of the advisory board will be contacted to identify any potentially relevant reviews that have yet to be identified.

### 4.3 Search strategy (non‐English language reviews)

The search strategy for non‐English language systematic reviews will mirror the search strategy used to identify primary research during Part I of the review.

### 4.4 Description of screening process

The screening process for English and non‐English language systematic reviews will mirror the respective screening processes used for screening primary research studies during Part. I. The abstract and full‐text screening tools used during Part II (see Appendix II) are based on the tools used in Part II, but they have been adapted for the screening of systematic reviews.

### 4.5 Review coding

The full text of included reviews will be coded using a process that aligns with that used in Part I. A separate data extraction and coding tool has been developed to guide the full text coding of reviews identified during Part II of the review (see Appendix III). This tool will capture relevant details about each individual review; descriptive information about the specific interventions, tools and/or approaches examined by each review; and the results of any quantitative or qualitative evidence synthesis or meta‐analysis.

A key difference between Part I and Part II relates to how different case management approaches will be coded. The coding of approaches in Part I uses pre‐defined codes that map onto the case management theory of change outlined in Section 1.2.6. In contrast, the coding of approaches in Part II uses a more open approach that is guided by the constituent parts of this theory of change, but which does not impose any pre‐defined codes. This more open approach is used on the basis that the assumptions underpinning interventions in other fields will not always map directly onto those underpinning counter‐radicalisation interventions.

A citation matrix (Pollock et al., 2021) will be used to identify the primary studies cited across each review, with separate citation matrices created for reviews relating to research Question 3 (i.e., reviews reporting on outcomes relating to countering violence) and research question 4 (i.e., reviews reporting on outcomes relating to implementation).

### 4.6 Assessing the quality of included reviews

Included reviews will be assessed using validated AMSTAR (A MeaSurement Tool to Assess systematic Reviews) quality assessment tool (Shea et al., 2017). The review team will use the updated AMSTAR 2 tool (Shea et al., 2017) as it can be used to assess the quality of systematic reviews including randomised and nonrandomised studies. The data extraction tool discussed above will incorporate the fifteen domains included in the AMSTAR 2 quality appraisal tool (Shea et al., 2017). The reviewers will independently assess each study using all fifteen domains, but the final assessment of quality will place greater weight on seven ‘critical domains’ as recommended by the tool's developers (Shea et al., 2017, p. 5). The domains are:

– Protocol registered before commencement of the review;
– Adequacy of the literature search;
– Justification for excluding individual studies;
– Risk of bias from individual studies being included in the review;
– Appropriateness of meta‐analytical methods (if study includes meta‐analysis);
– Consideration of risk of bias when interpreting the results of the review; and
– Assessment of presence and likely impact of publication bias.

Only reviews that are assessed as being of sufficient quality against these seven domains will be included in the main evidence synthesis. Included studies assessed as having a significant weakness relating to one or more of these domains will be analysed separately, and caveated accordingly. The review team will not reassess the quality of studies included within the individual reviews, but will instead capture the original review authors’ assessment of study quality at the data extraction stage. These quality assessments will be considered when interpreting the results of individual reviews, and reviews including studies assessed by the original author as being of poor quality will be caveated accordingly in the final review text.

### 4.7 Considering overlapping reviews

The citation matrices described in Section 4.5 will be used to identify the overlap between the reviews eligible for inclusion in the analysis of each research question. As Part II of the review does not seek to re‐analyse outcome data presented in the individual reviews (see below), overlapping reviews will be included in the review, and any overlap between reviews relevant to each research question will be made explicit by (a) outlining specific overlaps when presenting the analysis; and (b) including the citation matrix as an appendix to the final review.

### 4.8 Data analysis

Part II of the review will not seek to re‐analyse outcome data presented in the included reviews, but will instead present narrative summaries of the included reviews in line with Cochrane guidelines (Pollock et al., 2021). Separate narrative summaries will be presented for evidence relating to research Questions 3, 4 and 5 as outlined in Sections 4.8.1 and 4.8.2 below.

#### 4.8.1 Question 3 and 4: Evaluating effectiveness and implementation

Evidence relating to the effectiveness of tools and approaches in countering violence (Q3) and the process of implementing different tools and approaches (Q4) will be analysed and summarised separately. The analysis of both research questions will consist of four stages:

1. The different case management tools, approaches and stages being examined in each review will be identified, and a typology of different tools and approaches created. This typology will be created by JL, and reviewed by SM.
2. Primary and secondary outcome measures relevant to each research question will be identified, and a typology of different measures created.
3. Outcome data will then be organised thematically so that it is mapped against the case management tools, approaches and stages identified in Step 1.
4. Narrative summaries will be presented that discuss outcomes related to the different (a) case management approaches; (b) case management tools; and (c) stages of the case management process as outlined above. Each summary will include:
  a. An overview of how the specific tool/approach/stage is intended to work.
  b. A summary of data for each outcome identified for each tool/approach/stage (following Pollock et al. (2021) this will include effect estimates, 95% confidence intervals, and measures of heterogeneity (where relevant)).
  c. An assessment of the strength of evidence for each tool/approach/stage.

#### 4.8.2 Question 5: Transferability to counter‐radicalisation interventions

Answering Q5 as outlined above rests on developing a robust approach for identifying the most salient findings from research in the broader field of violence prevention for counter‐radicalisation work. This approach will be informed by existing studies that have discussed the comparability between these different fields. One of the most comprehensive discussions of this type is found in a RAND report exploring the ‘transferability and applicability of gang evaluation methodologies to counter‐violent radicalisation’. This report argues that lessons from gang‐related interventions are transferable to interventions seeking to counter radicalisation to violence because ‘[t]here are some key parallels between gangs and radicalisation, pertaining to demographics, recruitment, group dynamics, behaviour and desistance’ (Davies et al., 2017, p. 3). There are limits to such parallels, with comparative studies of violent extremists and gang members identifying demographic or experiential differences between these groups (e.g., Pyrooz et al., 2018; Windisch et al., 2022). However, these studies also point to opportunities for ‘expropriating prevention and intervention models designed to address gang problems and applying them to extremist groups’ (LaFree, 2018, p. 9) based on parallels in the processes by which individuals become engaged in, and disengage from, violent extremist organisations and gangs (LaFree, 2018).

In line with this body of research, the transferability of research examining tools and approaches seeking to counter other forms of violence will be assessed by asking the following questions of the evidence collected from studies included in Part II of the review. These questions have been informed by a checklist for assessing the transferability of the findings of systematic reviews to end‐users developed by Munthe‐Kaas et al. (2020):

– *Demographics/Population*: How does the profile of the population‐of‐interest compare to what is known about the population(s) supported through case management interventions seeking to counter radicalisation to violence (in terms of demographics; presence of comparable risk or protective factors etc.)?
– *Engagement*: Are the processes by which individuals become involved in the form of violence targeted by the tool/approach comparable to processes of radicalisation?
– *Disengagement*: Are the processes by which individuals disengage from the form of violence targeted by the tool/approach comparable to the processes by which individuals disengage from violent extremism?
– *Intervention*: How comparable is the tool/approach to those used to counter radicalisation to violence (in terms of, e.g., the risk/protective factors targeted; the different types of support provided; the context of delivery etc.)?
– *Feasibility*: Could the tools and approaches used feasibly be applied to interventions seeking to counter radicalisation to violence? Why/why not?

Assessing the transferability of tools and approaches in relation to the domains of *engagement* and *disengagement* is likely to be particularly challenging given that a) there is no single process by which individuals become engaged in, or disengage from, violent extremism; and b) these individualised processes of engagement and disengagement will not always directly map onto the processes by which individuals become involved in, and desist from, other forms of violence. However, models of engagement in, and disengagement from, violent offending more broadly (Cherney et al., 2021) and larger scale collective violence (Richards, 2017) share some common assumptions with dominant models of violent extremism engagement and disengagement. In turn, tools and approaches identified in Part II will be assessed as being transferable to counter‐radicalisation when they reflect these shared assumptions as reflected in the counter‐radicalisation theory of change outlined in Section 1.2.6, whereby processes of engagement and disengagement are understood as being:

– *Individualised* (even in the case of collective violence);
– Shaped by the intersection of different push and pull factors or *drivers* that:
  ○ operate at different levels of analysis (i.e., micro/meso/exo/macro)
  ○ are specific to the individual and/or the context in which they operate;
– Underpinned by specific *mechanisms* of change that are:
  ○ Informed by the presence (or absence) of specific risk/protective factors.
  ○ Reliant on agency (i.e., engagement in violence is an active choice; and long‐term disengagement rests on an individual wanting to desist);
– Are marked by specific attitudinal, motivational or behavioural *outcomes*;
– Are dependent on *contextual factors* that can inhibit or facilitate these processes.

### 4.9 Roles and responsibilities

**Content**: Marsden, Lewis, Cherney, Zeuthen.

**Systematic review methods**: Marsden, Lewis, Cherney, Zeuthen.

**Statistical analysis**: Brandsch.

**Information retrieval**: Marsden, Lewis, Zeuthen, Lubrano, Bélanger, Zubareva.

### 4.10 Potential conflicts of interest

Adrian Cherney has been involved in the evaluation of case‐managed programs targeting radicalised individuals in Australia and the publication of papers that are likely to be included in this review. However, Cherney will not be involved in the screening or coding of studies.

### 4.11 Preliminary timeframe

We plan to submit the final review by 20th February 2023.

## Supporting information

Supporting information.

## Footnotes

1 Whilst official statistics show that the number of Channel referrals has fallen from peak recorded in the wake of the Duty's introduction (Home Office, 2020), referral levels are still above pre‐2015 levels.

2 Part I of the review will include interventions using alternative terms to conceptualise ‘radicalisation to violence’ such as violent extremism; terrorism; and political violence (Schmid, 2013).

3 When working with individual clients, case management interventions will often engage with people in the client's social environment, including family and community members, with such engagement often being seen as crucial to positive outcomes (Cherney, 2021). Interventions that work with broader communities are included when such engagement is used as part of a tailored package of interventions for a specific individual client. However, broader community‐based work, such as interventions that seek to build resilience amongst whole communities, are not included in this systematic review.

4 The categorisations of PsycNet, Scopus, Web of Science and DOAJ as either principal or supplementary resources are taken directly from Gusenbauer and Haddaway's (2020) analysis. The categorisation of the remaining resources is based on each database's search functionality and coverage.

5 Available at https://www.ephpp.ca/quality-assessment-tool-for-quantitative-studies/

6 Available at https://casp-uk.net/casp-tools-checklists.

7
https://www.campbellcollaboration.org/escalc/html/EffectSizeCalculator-Home.php

8
https://www.campbellcollaboration.org/what-is-a-systematic-review.html
