# Supplementary material for: PROTOCOL: Case management interventions seeking to counter radicalisation to violence: A systematic review of tools and approaches
Source: Campbell Syst Rev. 2023 Feb 5;19(1):e1301. doi: 10.1002/cl2.1301 (PMC9899618; doi:10.1002/cl2.1301)
Supplement: Supplementary file 1 — Supporting information. [file CL2-19-e1301-s001.docx]

**Appendix I: English Language Key Words**

Part I Key Words: Countering radicalisation to violence

| **Problem** | **Intervention** | **Outcome** |
| --- | --- | --- |
| radicali* OR  extremis* OR  terroris* OR  jihadi* OR  islamis* OR  salafi* OR  right-wing OR  “right wing” OR  extreme-right OR  “extreme right” OR  neo-nazi OR  far-right OR  “far right” OR  Nationalist* OR  “white supremacis*“ OR  left-wing OR  “left wing” OR  extreme-left OR  “extreme left” OR  anarch* OR  “single issue” OR  single-issue | **Intervention**  initiative* OR  interven* OR  program* OR  policy OR  policies OR  scheme* OR  treat* OR  approach* OR  model* OR  strateg* OR  method* OR  project* OR  practice* OR  **Tool**  instrument* OR  tool* OR  framework* OR  protocol* OR  guid* OR  scale* OR  system* OR  inventor* OR  metric* OR  template* OR  profile* OR  criteria OR  questionnaire* OR  **Referral & assessment**  refer* OR  assess* OR  **Planning & management**  “case plan*” OR  “case formulat*” OR  “management plan*” OR  “treatment plan*” OR  “support plan*” OR  “case manage*” OR  “risk manage*” OR  **Monitoring**  progress* OR  monitor* OR  supervis* OR  measur* OR | prevent* OR  reduc* OR  counter* OR  disengage* OR  rehab* OR  reintegrat* OR  re-integrat* OR  re-entry OR  reentry OR  desist* OR  recidivism OR  deradical* OR  de-radical* OR  exit* |

Part II Key Words: Countering related forms of violence

| **Problem** | **Intervention** | **Outcome** | **Data** |
| --- | --- | --- | --- |
| **Interpersonal**  violen* OR  assault* OR  “batter” OR  batters OR  battere* OR  coerc* OR  beat* OR  stalk* OR  rape OR  homicid* OR  murder* OR  kill* OR  “intimate terror*” OR  “domestic abuse*” OR  “sex* abuse*” OR  “sex* offen*” OR  **Collective**  gangs OR  “gang” OR  rebel* OR  insurgent* OR  fighter* OR  combatant* OR  guerrilla* OR  militant* OR  soldier* | **Intervention**  initiative* OR  interven* OR  program* OR  policy OR  policies OR  scheme* OR  treat* OR  approach* OR  model* OR  strateg* OR  method* OR  project* OR  practice* OR  **Tool**  instrument* OR  tool* OR  framework* OR  protocol* OR  guid* OR  scale* OR  system* OR  inventor* OR  metric* OR  template* OR  profile* OR  criteria OR  questionnaire* OR  **Referral & assessment**  refer* OR  assess* OR  **Planning & management**  “case plan*” OR  “case formulat*” OR  “management plan*” OR  “treatment plan*” OR  “support plan*” OR  “case manage*” OR  “risk manage*” OR  **Monitoring**  progress* OR  monitor* OR  supervis* OR  measur* OR | prevent* OR  reduc* OR  counter* OR  disengage* OR  rehab* OR  reintegrat* OR  re-integrat* OR  re-entry OR  reentry OR  desist* OR  recidivism OR  exit* OR  de-mobili* OR demobili* OR disarm* OR  de-radical* OR  deradical* | meta-analys*  review* |

**Appendix II: Screening tool**

Part I: Countering radicalisation to violence

| **Topic** | **Inclusion criteria Research Question 1** | **Inclusion criteria Research Question 2** | **Exclusion criteria** |
| --- | --- | --- | --- |
| Duplicate | N/A | | Duplicate title, author and year to be excluded. |
| Research design | Quantitative study using an experimental research design to report on primary or secondary research data.  OR  Quantitative study using a stronger quasi-experimental research design to report on primary or secondary research data. | Quantitative study using an experimental, quasi-experimental or non-experimental research design to report on primary or secondary data.  OR  Qualitative study using any research design to report on primary or secondary data. | Study does not meet inclusion criteria for either Q1 or Q2. |
| Comparator condition | Treatment as usual OR Alternative treatment OR  No treatment | N/A | Question 1  Study does not use an eligible comparator condition.  No exclusion criteria for Question 2. |
| Individual focus | Intervention delivered to individual or tool that is individually-focused. | | Intervention delivered to collective or group. |
| Case management intervention, tool or approach. | **Intervention:** Examination of case management intervention or a stage in the case management process  OR  **Tool:** Examination of tool(s) used within case management process.  OR  **Approach:** Examination of approach(es) underpinning the case management process. | | Absence of case management intervention, tool or approach. |
| Radicalisation to violence | Intervention seeking to tackle cognitive or behavioural radicalisation/ deliver relevant primary or secondary outcomes:  **Primary:** preventing engagement in violent extremism or promoting disengagement from / preventing re-engagement in violent extremism.  **Secondary:** reduction in risk factors seen to contribute to radicalisation/ strengthening of strengths and/ or protective factors seen to contribute to resilience. | | Not explicitly focused on tackling cognitive or behavioural radicalisation |
| Effectiveness/ Implementation | Study examines the effectiveness of an intervention, tool or approach in delivering intervention outcomes | Study examines the process of implementing an intervention, tool or approach. | No discussion of either element. |

Part II: Countering other forms of violence

| **Topic** | **Inclusion criteria Research Question 3** | **Inclusion criteria Research Question 4** | | **Inclusion criteria Research Question 5** | | **Exclusion criteria** |
| --- | --- | --- | --- | --- | --- | --- |
| Duplicate | N/A | | | | | Duplicate title, author and year to be excluded. |
| Research design | Systematic reviews or meta-analyses | | | | | Not a systematic review or a meta-analysis |
| Individual focus | Intervention delivered to individual or tool that is individually-focused. | | | | | Intervention delivered to collective or group. |
| Case management intervention, tool or approach. | **Intervention:** Examination of case management intervention or stage in the case management process  OR  **Tool:** Examination of tool(s) used within case management process.  OR  **Approach:** Examination of approach(es) underpinning the case management process. | | | | | No discussion of case management intervention, tool or approach. |
| Violence | Intervention seeking to tackle individual involvement in violent behaviour of any type (e.g. domestic violence, gang-related violence, sexual violence)/ deliver relevant primary or secondary outcomes:  **Primary:** preventing individuals’ engagement in violence or promoting disengagement from violence/ preventing violent recidivism.  **Secondary:** reduction in risk factors seen to contribute violent behaviour/ strengthening of protective factors seen to contribute to resilience. | | | | | Interventions that do not specifically target violence. |
| Effectiveness/ Implementation | Study examines effectiveness of an intervention, tool or approach in delivering intervention outcomes | | Study examines the process of implementing intervention, tool or approach | | Study examines effectiveness of intervention, tool or approach in delivering intervention outcomes  OR  Study examines the process of implementing intervention, tool or approach. | No discussion of either effectiveness/ implementation. |
| Methods  (Full-text screening only) | The methodology used meets the following criteria:  - Authors must specify clear inclusion and exclusion criteria, and offer some justification.  - Authors must use explicit search strategy, specifying:  The different strategies used to identify research (e.g., keyword searches of academic databases; citation searching; reviewing grey literature, etc.).  The specific sources used to identify literature.  The process of screening studies.  The number of records identified in initial searches.  The number of unique studies included in the review.  - Authors must have conducted a systematic coding and analysis of included studies.  The methods of coding and analysis should be clearly outlined, and justified.  Where relevant, methods used to conduct meta-analysis must be specified. | | | | | Does not meet the specified criteria |

**Appendix III: Data extraction and coding framework**

1. Part I: Countering radicalisation to violence

| **Category** | **Field** | **Type** |
| --- | --- | --- |
| **Study**  **Information** | Author and year | Free text – format: Surname(s) (Year) |
|  | Title | Free text – study title |
|  | Source type | Pre-defined codes (single code)   - Journal article - Book - Book chapter - Think tank report - Government/ public agency report - Dissertation/ thesis - Other (specify) |
|  | Geographical focus | Free text – country where study conducted |
|  | Language | Free text – language |
| **Methods** | Type of research | Pre-defined codes (single code)   - Quantitative - Qualitative - Mixed methods |
|  | Quantitative  Research design | Pre-defined codes (single code)   - Experimental (specify) - Strong quasi-experimental (specify) - Weaker quasi experimental (specify) - Other (specify) - N/A |
|  | Qualitative Research Design | - Free text – research design. - N/A |
|  | Comparator condition | Pre-defined codes   - Treatment as usual - Alternative treatment - No treatment - Wait list - N/A |
|  | Randomisation | Pre-defined codes:   - Randomised - Quasi-randomised - Non-randomised - Other (specify) |
|  | Description of data collection and analysis | Free text – basic description of data collection and data analysis approach. |
|  | Population of study | Pre-defined codes (multi-code if needed)   - Practitioners - Clients - Other (specify) |
|  | Sample | Free text – description of sample. Specify:   - Sample demographics (if available) - Eligibility criteria - Any sub-groups analysed - Any other pertinent information. |
|  | Evidence relevant to research objectives | Pre-defined codes (multi-code if needed)   - Impact on radicalisation to violence - Process of implementation |
| **Intervention Details^[[1]](#footnote-1)^** | Focus | Pre-defined codes (multi-code if needed):   - Intervention(s) as-a-whole - Specific tool(s) (e.g. risk assessment) - Specific Approach(es) (e.g., RNR, strengths-based, etc.) |
|  | **Intervention Details I:**  Intervention name | Free text: name of intervention  N/A |
|  | **Intervention Details II:**  Intervention description | Free text: basic description of intervention  N/A |
|  | **Tool Details I:**  Tool name | Free text: name of tool  N/A |
|  | **Tool Details II:**  Tool description | Free text: basic description of tool  N/A |
|  | **Approach Details I:**  Description of approach | Free text: implicit or explicit theory of change of intervention/ tools examined. |
|  | **Approach Details II:**  Theory of change components | Pre-defined codes.  Answer all (multi-code if needed):  Drivers (I): Type of Driver   - Risk Factors - Protective Factors - Don’t know   Drivers (II): Domains   - Socio-demographic - Attitudinal - Psychological/ Personality - Experiential - Criminogenic - Don’t know   Drivers (III) Level of analysis   - Micro - Meso - Exo - Macro - Don’t know   Mechanisms   - Strengths-based mechanisms - Risk-oriented mechanisms - Don’t know   Progress/ Outcome Measures (I)   - Attitudinal - Motivational - Behavioural - Don’t know   Progress/ Outcome Measures (II)   - Reduction in risk factors - Development of protective factors - Building of strengths or skills - Don’t know |
|  | Stage of case management | Pre-defined codes (multi-code if needed):   - Identification/ referral - Risk assessment - Case planning - Implementation - Monitoring/ evaluation - Transition/ exit - All (single code) - Not specified (single code) |
| **Intervention Context** | Country of delivery | Free text – country of delivery. |
|  | Population | Pre-defined codes (multi-code if needed)   - Secondary prevention - Tertiary prevention |
|  | Context | Free text - brief description of intervention context including any implementation and moderator factors as listed in Section 1.2.6. |
|  | Delivery agents | Free text – list the different types of practitioners who deliver intervention. |
|  | Transferability across contexts | Free text – qualitative assessment of extent to which findings would be relevant outside specific context examined in study. |
|  | | |
| **Meta-Analysis Research Question 1: Countering Radicalisation to Violence** | | |
| **Progress and Outcome Measures** | Tools used to monitor client-level progress | Free text – description of how client-level progress is captured (if relevant) |
|  | Client-level progress measures | Free text – list of client-level progress measures (if relevant) |
|  | Tools used to measure client-level outcomes | Free text – description of how client-level outcome measures are captured (if relevant) |
|  | Client-level intervention outcomes (Primary) | Free text – list/ describe primary outcomes (e.g. prevent radicalisation; promote disengagement etc.) |
|  | Client-level intervention outcomes (Secondary) | Free text – list/ describe secondary outcomes (e.g. risk/ protective factors) |
| **Measurement of Progress and Outcomes^[[2]](#footnote-2)^**  ***To be completed for all measures identified above*** | Type of measure | Pre-defined codes   - Progress - Outcome |
|  | Description of measure | Free text – name and description of the progress/ outcome measure. |
|  | Measurement tool | Free text – name/ type of tool used to capture measure (e.g. risk assessment framework; case notes, etc.) |
|  | Source of data used in original analysis. | Pre-defined codes   - Self-report - Observation - Official source - Interview - Other (specify) |
|  | Psychometric properties of the measurement tool (e.g., reliability, validity, etc.)? | Free text – description of properties. |
|  | Type of respondent. | Free text – description of the type of respondent from which data was collected (e.g. client, practitioner etc.) |
|  | Time-points at which measurement taken. | Free text - time-points at which measure collected (e.g. pre/ post intervention) |
|  | Was data collected in same way for treatment and comparison condition? | Pre-defined codes   - Yes - No (specify) - Unclear |
|  | Are there any raw differences (i.e. significant or non-significant) differences between control and treatment group? | Pre-defined codes   - Yes – results favour treatment - Yes – results favour comparison - No - Unclear |
|  | Direction of outcome change | Pre-defined codes   - Positive - Negative - Mixed (specify) - Unclear |
|  | Statistically significant differences for outcome | Pre-defined codes   - Yes - No - Not tested - Unclear |
|  | Study author(s)’ conclusions | Free text – outline conclusions drawn in original study about this outcome. |
|  | Coder’s conclusions | Free text – does outcome data suggest that tool or approach is effective? Explain. |
| **Effect Size^[[3]](#footnote-3)^**  ***To be completed for all measures identified above*** | Page number | Free text – page number on which effect size reported. |
|  | Type of effect captured | Pre-defined codes   - Post-intervention only - Pre-intervention & post-intervention - Follow-up after initial post-intervention measurement. |
|  | Timeframe captured | Free text for all options.   - Minimum time - Maximum time - Mean time - Fixed (single code) |
|  | How effect size is captured | Pre-defined codes   - Reported in document - Calculated by research team |
|  | *If reported in document*  Effect size | Free text – reported effect size. |
|  | *If calculated by researchers*  Data and calculations used to calculate effect size | Free text – data used, calculations and final effect size calculated. |
| **Overall Assessment** | Coder’s overall assessment of the tool or approach in regard to effectiveness at countering radicalisation to violence | Pre-defined codes   - Effective (specify) - Promising (specify) - Mixed results (specify) - Ineffective (specify) - Unclear (specify) |

| **Meta-Analysis Research Question 2: Implementation**  **(Only for studies using experimental or stronger quasi-experimental designs)** | | |
| --- | --- | --- |
| **Measures of Implementation** | Implementation measures | Free text – list/ description of measures  N/A |
| **Measurement of Implementation^[[4]](#footnote-4)^**  ***To be completed for all measures identified above.*** | Description of measure | Free text – name and description of the progress/ outcome measure. |
|  | Source of data used in original analysis. | Pre-defined codes   - Self-report - Observation - Official source - Interview - Other (specify) |
|  | Type of respondent. | Free text – description of the type of respondent from which data was collected (e.g. client, practitioner etc.) |
|  | Time-points at which measurement taken. | Free text - time-points at which measure collected (e.g. pre/ post intervention) |
|  | Was data collected in same way for treatment and comparison condition? | Pre-defined codes   - Yes - No (specify) - Unclear |
|  | Are there any raw differences (i.e. significant or non-significant) differences between control and treatment group? | Pre-defined codes   - Yes – results favour treatment - Yes – results favour comparison - No - Unclear |
|  | Direction of outcome change | Pre-defined codes   - Positive - Negative - Mixed (specify) - Unclear |
|  | Statistically significant differences for outcome | Pre-defined codes   - Yes - No - Not tested - Unclear |
|  | Study author(s)’ conclusions | Free text – outline conclusions drawn in original study about this outcome. |
|  | Coder’s conclusions | Free text – does outcome data suggest that tool or approach is effective? |
| **Effect Size^[[5]](#footnote-5)^**  ***To be completed for all measures identified above*** | Page number | Free text – page number on which effect size reported. |
|  | Type of effect captured | Pre-defined codes   - Post-intervention only - Pre-intervention & post-intervention - Follow-up after initial post-intervention measurement. |
|  | Timeframe captured | Free text for all options.   - Minimum time - Maximum time - Mean time - Fixed (single code) |
|  | How effect size is captured | Pre-defined codes   - Reported in document - Calculated by research team |
|  | *If reported in document*  Effect size | Free text – reported effect size. |
|  | *If calculated by researchers*  Data and calculations used to calculate effect size | Free text – data used, calculations and final effect size calculated. |
| **Overall Assessment** | Coder’s overall assessment of the tool or approach in regard to impact on professional practice | Pre-defined codes   - Positive effect (specify) - Promising effect (specify) - Mixed results (specify) - No effect (specify) - Negative effect (specify) - Unclear (specify) |

| **Other Synthesis Research Question 2: Implementation**  **(Only for studies qualitative or weaker quantitative designs)** | | | | | | |
| --- | --- | --- | --- | --- | --- | --- |
| **Q2a – Process of Implementation**  **(if relevant)** | | | Measure of implementation | Free text – list/ describe measures | | |
|  |  |  | Description of measure | Free text – name and description of the progress/ outcome measure. | | |
|  |  |  | Source of data used in original analysis. | Pre-defined codes   - Self-report - Observation - Official source - Interview   Other (specify) | | |
|  |  |  | Type of respondent. | Free text – description of the type of respondent from which data was collected (e.g. client, practitioner etc.) | | |
|  |  |  | Implemented in way expected? | Free text – description of extent to which implemented in way expected. | | |
|  |  |  | Evidence in support specific intervention, tool, or approach | Free text – positive research findings (page number) | | |
|  |  |  | Evidence opposed to specific intervention, tool, or approach | Free text – negative research findings (page number) | | |
|  |  |  | Study author(s)’ conclusions | Free text – outline conclusions drawn in original study about process. | | |
|  |  |  | Coder’s conclusions | Free text –coder conclusions about process of implementation. | | |
| **Q2b – Implementation Factors/ Moderators**  **(if relevant)** | | | Implementation factors/ moderators. | Free text – describe factors/ moderators discussed. | | |
|  |  |  | Source of data used in original analysis. | Pre-defined codes   - Self-report - Observation - Official source - Interview   Other (specify) | | |
|  |  |  | Type of respondent. | Free text – description of the type of respondent from which data was collected (e.g. client, practitioner etc.) | | |
|  |  |  | Evidence of implementation factors/ moderators having a positive effect on process. | Free text – positive findings | | |
|  |  |  | Evidence of implementation factors/ moderators having a negative effect on process. | Free text – negative findings | | |
|  | | | Study author(s)’ conclusions | Free text – outline conclusions drawn in original study about factor(s) | | |
|  |  |  | Coder’s conclusions | Free text –coder conclusions about factor/ moderator. | | |
|  | | | | | | |
| **Risk of Bias: Non-Randomised Studies: ROBINS-I^[[6]](#footnote-6)^**  **(Research Question 1 and 2 – If Relevant)** | | | | | | |
| **ROBINS-I (I)**  **Bias due to Cofounding** | | 1.1. Is there potential for confounding of the effect of intervention in this study? | | | Yes  Probably Yes  Probably No  No  *If No/ Probably No, risk of bias is low for this domain and no further signalling questions are required..* | |
|  |  | *Only if Yes/ Probably Yes at 1.1.*  1.2. Was the analysis based on splitting participants’ follow up time according to intervention received? | | | N/A  Yes  Probably Yes  Probably No  No  No Information | |
|  |  | *Only if Yes/ Probably Yes at 1.2*  1.3. Were intervention discontinuations or switches likely to be related to factors that are prognostic for the outcome? | | | N/A  Yes  Probably Yes  Probably No  No  No Information | |
|  |  | *Only if Yes/ Probably Yes at 1.1.*  1.4. Did the authors use an appropriate analysis method that controlled for all the important confounding areas? | | | N/A  Yes  Probably Yes  Probably No  No  No Information | |
|  |  | *Only if Yes/ Probably Yes at 1.4.*  1.5. Were confounding areas that were controlled for measured validly and reliably by the variables available in this study? | | | N/A  Yes  Probably Yes  Probably No  No  No Information | |
|  |  | *Only if Yes/ Probably Yes at 1.1.*  1.6. Did the authors control for any post-intervention variables? | | | N/A  Yes  Probably Yes  Probably No  No  No Information | |
|  |  | *Only if Yes/ Probably Yes at 1.3*  1.7. Did the authors use an appropriate analysis method that adjusted for all the important confounding areas and for time varying confounding? | | | N/A  Yes  Probably Yes  Probably No  No  No Information | |
|  |  | *If Yes/ Probably Yes at 1.7*  1.8. Were confounding areas that were adjusted for measured validly and reliably by the variables available in this study? | | | N/A  Yes  Probably Yes  Probably No  No  No Information | |
|  |  | Risk of Bias for Domain | | | Low  Moderate  Serious  Critical  No Information | |
| **ROBINS-I (II)**  **2. Bias in selection of participants into the study** | | 2.1. Was selection of participants into the study (or into the analysis) based on participant characteristics observed after the start of intervention? | | | Yes  Probably Yes  Probably No (Go to 2.4)  No (Go to 2.4)  No Information | |
|  |  | *Only if Yes/ Probably Yes at 2.1*  2.2. Were the post-intervention variables that influenced selection likely to be associated with intervention? | | | N/A  Yes  Probably Yes  Probably No  No  No Information | |
|  |  | *If Yes/ Probably Yes at 2.2*  2.3. Were the post-intervention variables that influenced selection likely to be influenced by the outcome or a cause of the outcome? | | | N/A  Yes  Probably Yes  Probably No  No  No Information | |
|  |  | 2.4. Do start of follow-up and start of intervention coincide for most participants? | | | Yes  Probably Yes  Probably No  No  No Information | |
|  |  | *If Yes /Probably Yes to 2.2 & 2.3, or*  *If No/ Probably No to 2.4*  2.5. Were adjustment techniques used that are likely to correct for the presence of selection biases? | | | N/A  Yes  Probably Yes  Probably No  No  No Information | |
|  |  | Risk of Bias Judgement for Domain | | | Low  Moderate  Serious  Critical  No Information | |
| **ROBINS-I (III)**  **3. Bias in classification of interventions** | | 3.1. Were intervention groups clearly defined? | | | Yes  Probably Yes  Probably No  No  No Information | |
|  |  | 3.2. Was the information used to define intervention groups recorded at the start of the intervention? | | | Yes  Probably Yes  Probably No  No  No Information | |
|  |  | 3.3. Could classification of intervention status have been affected by knowledge of the outcome or risk of the outcome? | | | Yes  Probably Yes  Probably No  No  No Information | |
|  |  | Risk of Bias for Domain | | | Low  Moderate  Serious  Critical  No Information | |
| **ROBINS-I (IV)**  **4. Bias as a result of departures from intended interventions** | | 4.1. Were there deviations from the intended intervention beyond what would be expected in usual practice? | | | Yes  Probably Yes  Probably No  No  No Information | |
|  |  | *If Yes/ Probably Yes to 4.1.*  4.2. Were these deviations from intended intervention unbalanced between groups and likely to have affected the outcome? | | | N/A  Yes  Probably Yes  Probably No  No  No Information | |
|  |  | 4.3. Were important cointerventions balanced across intervention groups? | | | Yes  Probably Yes  Probably No  No  No Information | |
|  |  | 4.4. Was the intervention implemented successfully for most participants? | | | Yes  Probably Yes  Probably No  No  No Information | |
|  |  | 4.5. Did study participants adhere to the assigned intervention regimen? | | | Yes  Probably Yes  Probably No  No  No Information | |
|  |  | *If No/ Probably No to 4.3, 4.4 or 4.5.*  4.6. Was an appropriate analysis used to estimate the effect of starting and adhering to the intervention? | | | N/A  Yes  Probably Yes  Probably No  No  No Information | |
|  |  | Risk of Bias Judgement for Domain | | | Low  Moderate  Serious  Critical  No Information | |
| **ROBINS-I (V)**  **5. Bias as a result of missing data** | | 5.1. Were outcome data available for all, or nearly all, participants? | | | Yes  Probably Yes  Probably No  No  No Information | |
|  |  | 5.2. Were participants excluded due to missing data on intervention status? | | | Yes  Probably Yes  Probably No  No  No Information | |
|  |  | 5.3. Were participants excluded due to missing data on other variables needed for the analysis? | | | Yes  Probably Yes  Probably No  No  No Information | |
|  |  | *If No/ Probably No to 5.1 or*  *If Yes/ Probably Yes to 5.2 or 5.3*  5.4. Are the proportion of participants and reasons for missing data similar across interventions? | | | N/A  Yes  Probably Yes  Probably No  No  No Information | |
|  |  | *If No/ Probably No to 5.1 or*  *If Yes/ Probably Yes to 5.2 or 5.3*  5.5. Is there evidence that results were robust to the presence of missing data? | | | N/A  Yes  Probably Yes  Probably No  No  No Information | |
|  |  | Risk of Bias Judgement for Domain | | | Low  Moderate  Serious  Critical  No Information | |
| **ROBINS-I (VI)**  **6. Bias in measurement of outcomes** | | 6.1. Could the outcome measure have been influenced by knowledge of the intervention received? | | | Yes  Probably Yes  Probably No  No  No Information | |
|  |  | 6.2. Were outcome assessors aware of the intervention received by study participants? | | | Yes  Probably Yes  Probably No  No  No Information | |
|  |  | 6.3. Were the methods of outcome assessment comparable across intervention groups? | | | Yes  Probably Yes  Probably No  No  No Information | |
|  |  | 6.4. Were any systematic errors in measurement of the outcome related to intervention received? | | | Yes  Probably Yes  Probably No  No  No Information | |
|  |  | Risk of Bias Judgement for Domain | | | Low  Moderate  Serious  Critical  No Information | |
| **ROBINS-I (VII)**  **7. Bias in selection of the reported result** | | 7.1. Is the reported effect estimate likely to be selected, on the basis of the results, from multiple outcome measurements within the outcome domain? | | | Yes  Probably Yes  Probably No  No  No Information | |
|  |  | 7.2. Is the reported effect estimate likely to be selected, on the basis of the results, from multiple analyses of the intervention–outcome relationship? | | | Yes  Probably Yes  Probably No  No  No Information | |
|  |  | 7.3. Is the reported effect estimate likely to be selected, on the basis of the results, from different subgroups? | | | Yes  Probably Yes  Probably No  No  No Information | |
|  |  | Risk of Bias Judgement for Domain | | | Low  Moderate  Serious  Critical  No Information | |
| **Overall Risk of Bias** | | Overall risk of bias | | | Low  Moderate  Serious  Critical | |
| **Risk of Bias: Randomised Studies: RoB 2^[[7]](#footnote-7)^**  **(Research Question 1 and 2 – If Relevant)** | | | | | | |
| **ROB 2 (I)**  **Bias arising from the randomisation process** | 1.1 Was the allocation sequence random? | | | | | Yes  Probably Yes  Probably No  No  No Information |
|  | 1.2 Was the allocation sequence concealed until participants were enrolled and assigned to interventions? | | | | | Yes  Probably Yes  Probably No  No  No Information |
|  | 1.3 Did baseline differences between intervention groups suggest a problem with the randomisation process? | | | | | Yes  Probably Yes  Probably No  No  No Information |
|  | Risk of Bias for Domain | | | | | High  Low  Some Concerns |
| **ROB 2 (II)**  **Bias due to deviations from intended interventions** | 2.1 Were participants aware of their assigned intervention during the trial? | | | | | Yes  Probably Yes  Probably No  No  No Information |
|  | 2.2 Were carers and people delivering the interventions aware of participants’ assigned intervention during the trial? | | | | | Yes  Probably Yes  Probably No  No  No Information |
|  | If Yes/ Probably Yes /No Information to 2.1 or 2.2  2.3. Were there deviations from the intended intervention that arose because of the trial context? | | | | | N/A  Yes  Probably Yes  Probably No  No  No Information |
|  | If Yes/ Probably Yes to 2.3  2.4. Were these deviations likely to have affected the outcome? | | | | | N/A  Yes  Probably Yes  Probably No  No  No Information |
|  | If Yes/ Probably Yes/ No Information to 2.4.  2.5. Were these deviations from intended intervention balanced between groups? | | | | | N/A  Yes  Probably Yes  Probably No  No  No Information |
|  | 2.6 Was an appropriate analysis used to estimate the effect of assignment to intervention? | | | | | Yes  Probably Yes  Probably No  No  No Information |
|  | If No /Probably No/ No Information to 2.6.  2.7 Was there potential for a substantial impact (on the result) of the failure to analyse participants in the group to which they were randomised? | | | | | N/A  Yes  Probably Yes  Probably No  No  No Information |
|  | Risk of Bias for Domain | | | | | High  Low  Some Concerns |
| **RoB 2 (III)**  **Bias due to missing outcome data** | 3.1 Were data for this outcome available for all, or nearly all, participants randomised? | | | | | Yes  Probably Yes  Probably No  No  No Information |
|  | If No /Probably No /No Information to 3.1.  3.2 Is there evidence that the result was not biased by missing outcome data? | | | | | N/A  Yes  Probably Yes  Probably No  No |
|  | If No /Probably No to 3.2.  3.3 Could missingness in the outcome depend on its true value? | | | | | N/A  Yes  Probably Yes  Probably No  No  No Information |
|  | If Yes/ Probably Yes /No Information to 3.3.  3.4 Is it likely that missingness in the outcome depended on its true value? | | | | | N/A  Yes  Probably Yes  Probably No  No  No Information |
|  | Risk of Bias for Domain | | | | | High  Low  Some Concerns |
| **RoB 2 (IV)**  **Bias in measurement of outcome** | 4.1 Was the method of measuring the outcome inappropriate? | | | | | Yes  Probably Yes  Probably No  No  No Information |
|  | 4.2 Could measurement or ascertainment of the outcome have differed between intervention groups? | | | | | Yes  Probably Yes  Probably No  No  No Information |
|  | If No /Probably No / No Information to 4.1 & 4.2.  4.3 Were outcome assessors aware of the intervention received by study participants? | | | | | Yes  Probably Yes  Probably No  No  No Information |
|  | If Yes /Probably Yes/ No Information to 4.3.  4.4 Could assessment of the outcome have been influenced by knowledge of intervention received? | | | | | N/A  Yes  Probably Yes  Probably No  No  No Information |
|  | If Yes /Probably Yes/ No Information to 4.4.  4.5 Is it likely that assessment of the outcome was influenced by knowledge of intervention received? | | | | | N/A  Yes  Probably Yes  Probably No  No  No Information |
|  | Risk of Bias for Domain | | | | | High  Low  Some Concerns |
| **RoB 2 (V)**  **Bias in selection of the reported result** | 5.1 Were the data that produced this result analysed in accordance with a prespecified analysis plan that was finalised before unblinded outcome data were available for analysis? | | | | | Yes  Probably Yes  Probably No  No  No Information |
|  | 5.2. Is the numerical result being assessed likely to have been selected, on the basis of the results, from: multiple eligible outcome measurements (eg, scales, definitions, time points) within the outcome domain? | | | | | Yes  Probably Yes  Probably No  No  No Information |
|  | 5.3. Is the numerical result being assessed likely to have been selected, on the basis of the results, from: multiple eligible analyses of the data? | | | | | Yes  Probably Yes  Probably No  No  No Information |
|  | Risk of Bias Judgement for Domain | | | | | Low  Moderate  Serious  Critical  No Information |
| **Overall Risk of Bias** | Risk of Bias | | | | | High  Low  Some Concerns |

| **Risk of Bias: EPHPP Quality Assessment Tool^[[8]](#footnote-8)^**  **(Research Question 2 Only – If Relevant)** | | |
| --- | --- | --- |
| **A Selection Bias** | Are the individuals selected to participate in the study likely to be representative of the target population? | 1 Very likely  2 Somewhat likely  3 Not likely  4 Can’t tell |
|  | What percentage of selected individuals agreed to participate? | 1 80 - 100%  2 60 – 79%  3 less than 60%  4 Not applicable  5 Can’t tell |
|  | Overall rating for section | 1 Strong  2 Moderate  3 Weak |
| **B Study Design** | Indicate the study design | 1 Randomized controlled trial  2 Controlled clinical trial  3 Cohort analytic (two group pre + post)  4 Case-control  5 Cohort (one group pre + post (before & after))  6 Interrupted time series  7 Other (specify) |
|  | Was the study described as randomized? | 1 No (Go to component C)  2 Yes |
|  | If Yes  Was the method of randomization described? | 1 No  2 Yes |
|  | If Yes  Was the method appropriate? | 1 No  2 Yes |
|  | Overall rating for section | 1 Strong  2 Moderate  3 Weak |
| **C Confounders** | Were there important differences between groups prior to the intervention?  Examples of confounders:  1 Race; 2 Sex; 3 Marital status/family; 4 Age;  5 SES (income or class); 6 Education; 7 Health status; 8 Pre-intervention score on outcome measure. | 1 Yes  2 No  3 Can’t tell |
|  | If Yes.  Indicate the percentage of relevant confounders that were controlled (either in the design (e.g. stratification, matching) or analysis)? | 1 80 – 100% (most)  2 60 – 79% (some)  3 Less than 60% (few or none)  4 Can’t Tell |
|  | Overall rating for section | 1 Strong  2 Moderate  3 Weak |
| **D BLINDING** | Was (were) the outcome assessor(s) aware of the intervention or exposure status of participants? | 1 Yes  2 No  3 Can’t tell |
|  | Were the study participants aware of the research question? | 1 Yes  2 No  3 Can’t tell |
|  | Overall rating for section | 1 Strong  2 Moderate  3 Weak |
| **E DATA COLLECTION METHODS** | Were data collection tools shown to be valid? | 1 Yes  2 No  3 Can’t tell |
|  | Were data collection tools shown to be reliable? | 1 Yes  2 No  3 Can’t tell |
|  | Overall rating for section | 1 Strong  2 Moderate  3 Weak |
| **F WITHDRAWALS AND DROP- OUTS** | Were withdrawals and drop-outs reported in terms of numbers and/or reasons per group? | 1 Yes  2 No  3 Can’t tell  4 Not Applicable (i.e. one time surveys or interviews) |
|  | Indicate the percentage of participants completing the study. (If the percentage differs by groups, record the lowest). | 1 80 -100%  2 60-79%  3 less than 60%  4 Can’t tell  5 Not Applicable (i.e. Retrospective case-control) |
|  | Overall rating for section | 1 Strong  2 Moderate  3 Weak  Not applicable |
| **G INTERVENTION INTEGRITY** | What percentage of participants received the allocated intervention or exposure of interest | 1 80 -100%  2 60-79%  3 less than 60%  4 Can’t tell |
|  | Was the consistency of the intervention measured? | 1 Yes  2 No  3 Can’t tell |
|  | Is it likely that the subjects received an unintended intervention (contamination or co-intervention) that may the results? | 1 Yes  2 No  3 Can’t tell |
| **H ANALYSES** | Indicate the unit of allocation (single code) | Community  Organization/institution  Practice/office  Individual |
|  | Indicate the unit of analysis (single code) | Community  Organization/institution  Practice/office  Individual |
|  | Are the statistical methods appropriate for the study design? | 1 Yes  2 No  3 Can’t tell |
|  | Is the analysis performed by intervention allocation status (i.e. intention to treat) rather than the actual intervention received? | 1 Yes  2 No  3 Can’t tell |
| **GLOBAL RATING** | Overall quality | 1 STRONG (No WEAK ratings)  2 MODERATE (One WEAK rating)  3 WEAK (Two or more WEAK ratings) |

| **Assessment of Qualitative Research CASP Checklist^[[9]](#footnote-9)^**  **(Research Question 2 Only – If Relevant)** | | |
| --- | --- | --- |
| Question | Answer Options | Consider |
| Was there a clear statement of the aims of the research? | Yes  No  Can’t Tell | - What was the goal of the research; - Why it was thought important; - Its relevance. |
| Is a qualitative methodology appropriate? | Yes  No  Can’t Tell | - If the research seeks to interpret or illuminate the actions and/or subjective experiences of research participants; - Is qualitative research the right methodology for addressing the research goal. |
| Was the research design appropriate to address the aims of the research? | Yes  No  Can’t Tell | - If the researcher has justified the research design (e.g. have they discussed how they decided which method to use) |
| Was the recruitment strategy appropriate to the aims of the research? | Yes  No  Can’t Tell | - If the researcher has explained how the participants were selected; - If they explained why the participants they selected were the most appropriate to provide access to the type of knowledge sought by the study; - If there are any discussions around recruitment (e.g. why some people chose not to take part) |
| Was the data collected in a way that addressed the research issue? | Yes  No  Can’t Tell | - If the setting for the data collection was justified; - If it is clear how data were collected (e.g. focus group, semi-structured interview etc.); - If the researcher has justified the methods chosen; - If the researcher has made the methods explicit (e.g. for interview method, is there an indication of how interviews are conducted, or did they use a topic guide); - If methods were modified during the study. If so, has the researcher explained how and why; - If the form of data is clear (e.g. tape recordings, video material, notes etc.); - If the researcher has discussed saturation of data |
| Has the relationship between researcher and participants been adequately considered? | Yes  No  Can’t Tell | - If the researcher critically examined their own role, potential bias and influence during (a) formulation of the research questions (b) data collection, including sample recruitment and choice of location; - How the researcher responded to events during the study and whether they considered the implications of any changes in the research design. |
| Have ethical issues been taken into consideration? | Yes  No  Can’t Tell | - If there are sufficient details of how the research was explained to participants for the reader to assess whether ethical standards were maintained; - If the researcher has discussed issues raised by the study (e.g. issues around informed consent or confidentiality or how they have handled the effects of the study on the participants during and after the study); - If approval has been sought from the ethics committee. |
| Was the data analysis sufficiently rigorous? | Yes  No  Can’t Tell | - If there is an in-depth description of the analysis process; - If thematic analysis is used. If so, is it clear how the categories/themes were derived from the data; - Whether the researcher explains how the data presented were selected from the original sample to demonstrate the analysis process; - If sufficient data are presented to support the findings; - To what extent contradictory data are taken into account; - Whether the researcher critically examined their own role, potential bias and influence during analysis and selection of data for presentation. |
| Is there a clear statement of findings? | Yes  No  Can’t Tell | - If the findings are explicit; - If there is adequate discussion of the evidence both for and against the researcher’s arguments; - If the researcher has discussed the credibility of their findings (e.g. triangulation, respondent validation, more than one analyst); - If the findings are discussed in relation to the original research question |

1. Part II: Countering other forms of violence - Review coding

| **Category** | **Field** | **Type** |
| --- | --- | --- |
| **Study**  **Information** | Author and year | Free text – format: Surname(s) (Year) |
|  | Title | Free text – study title |
|  | Source type | Pre-defined codes (single code)   - Journal article - Book - Book chapter - Think tank report - Government/ public agency report - Dissertation/ thesis - Other (specify) |
|  | Geographical focus | Free text – country where study conducted |
|  | Language | Free text – language |
| **Methods** | Type of study | Pre-defined codes (multi-code if needed)   - Systematic review - Meta-analysis - Other synthesis (specify) |
|  | Research designs included | Pre-defined codes (multi-code if needed)   - Experimental (specify) - Strong quasi-experimental (specify) - Weaker quasi experimental (specify) - Other (specify) |
|  | Comparator conditions | Pre-defined codes   - Treatment as usual - Alternative treatment - No treatment - Wait list - Other (specify) - N/A |
|  | Randomisation | Pre-defined codes:   - Only randomised studies included - Non-randomised studies included |
|  | Research designs included | Free text – specific research designs. |
|  | Description of data collection and analysis | Free text – basic description of data collection and data analysis approach. Include inclusion and exclusion criteria. |
|  | Population of study | Pre-defined codes (multi-code if needed)   - Practitioners - Clients - Other (specify) |
|  | Number of studies included | Number |
|  | Evidence relevant to research objectives | Pre-defined codes (multi-code if needed)   - Impact on violence - Implementation |
| **Intervention Details^[[10]](#footnote-10)^** | Focus | Pre-defined codes (multi-code if needed)   - Intervention(s) as a whole - Specific Tool(s) (e.g. risk assessment) - Specific Approach(es) (e.g. RNR, strengths-based) |
|  | Type of violence (I) | Pre-defined codes (multi-code if needed)   - Interpersonal - Collective |
|  | Type of violence (II) | Free-text: focus of the intervention/ tool/ approach (e.g. gang-related; domestic violence; general violent offending etc.) |
|  | **Intervention Details I:**  Intervention name | Free text: name of intervention  N/A |
|  | **Intervention Details II:**  Intervention description | Free text: basic description of intervention  N/A |
|  | **Tool Details I:**  Tool name | Free text: name of tool  N/A |
|  | **Tool Details II:**  Tool description | Free text: basic description of tool  N/A |
|  | **Approach Details I:**  Intervention approach | Free text: implicit or explicit theory of change of intervention/ tools examined. |
|  | **Approach Details II:**  Theory of change components | Free-text  Answer all:  Drivers:  Describe drivers, considering:   - Does the intervention consider risk/ and or protective factors? - Does the intervention consider different types of driver (e.g. demographic, attitudinal etc.)? - Does the intervention consider factors at different levels of analysis?   Mechanisms:  Describe mechanisms, considering:   - Does the review explicitly examine a specific model of rehabilitation or some combination of models (e.g., RNR, strengths-based, GLM, etc.)? - Do assumptions (explicit or implicit) underpinning the intervention/ tool/ approach align with specific model(s) of rehabilitation?   Progress/ Outcome measures  Describe measures, considering:   - What types of measures are used (e.g. attitudinal, behavioural, etc.)? - How do they relate to intervention outcomes (e.g., do they relate to a reduction in risk, building protective factors, etc.)? |
|  | Stage of case management | Pre-defined codes (multi-code if needed)   - Identification/ referral - Risk assessment - Case planning - Implementation - Monitoring/ evaluation - Transition/ exit - All (single code) - Not specified (single code) |
| **Intervention Context** | Country of delivery | Free text – country of delivery. |
|  | Population | Pre-defined codes (multi-code if needed)   - Secondary prevention - Tertiary prevention |
|  | Context | Free text - brief description of intervention context including any implementation and moderator factors as listed in Section 1.2.6. |
|  | Delivery agents | Free text – list the different types of practitioners who deliver intervention. |
|  | Transferability across contexts | Free text – qualitative assessment of extent to which findings relevant outside of the specific context examined in study. |
| **Q 3. Progress and Outcome Measures** | Tools used to monitor client-level progress | Free text – description of how client-level progress are captured (if relevant) |
|  | Client-level progress measures | Free text – list of client-level progress measures (if relevant) |
|  | Tools used to measure client-level outcomes | Free text – description of how client-level outcome measures are captured (if relevant) |
|  | Client-level intervention outcomes  (Primary) | Free text – list/ describe primary outcomes (e.g. prevent radicalisation; promote disengagement/ deradicalisation etc.) |
|  | Client-level intervention outcomes  (Secondary) | Free text – list/ describe secondary outcomes (e.g. specific risk/ protective factors) |
| **Q4. Implementation Process**  **(if relevant)** | Qualitative data | Free text – description of key data presented on implementation. |
|  | Quantitative data. | Free text – present data from any quantitative analysis incl. meta-analysis.  Include: effect sizes, confidence intervals,  assessment of heterogeneity (if relevant). |
|  | Author Conclusions | Free text – author conclusions on process |
|  | Coder Conclusions | Free text – coder conclusions on process |
| **Q4.**  **Implementation Factors**  **(if relevant)** | Qualitative data | Free text – description of key data presented on implementation factors. |
|  | Quantitative data. | Free text – present data from any quantitative analysis incl. meta-analysis.  Include: effect sizes, confidence intervals,  assessment of heterogeneity (if relevant). |
|  | Author Conclusions | Free text – author conclusions on factors |
|  | Coder Conclusions | Free text – coder conclusions on factors |
| **Impact on violence**  **(if relevant)** | Qualitative synthesis of evidence | Free text – description of key data presented on effectiveness. |
|  | Results from quantitative analysis | Free text – present data from any quantitative analysis incl. meta-analysis.  Include: effect sizes, confidence intervals,  assessment of heterogeneity (if relevant). |
|  | Conclusions – are tools and approaches effective? | Free text – summary of conclusions. |
| **Transferability to countering radicalisation to violence** | Transferable lessons | Free text – qualitative assessment of transferability including discussion of key findings relevant to interventions seeking to counter radicalisation to violence. |
|  | | |
| **Quality Assessment – AMSTAR II** | | |
| **Quality –**  **AMSTAR 2^[[11]](#footnote-11)^** | Did the research questions and inclusion criteria for the review include the components of PICO? | Yes  No |
|  | Did the report of the review contain an explicit statement that the review methods were established prior to conduct of the review and did the report justify any significant deviations from the protocol? | Yes  Partial Yes  No |
|  | Did the review authors explain their selection of the study designs for inclusion in the review? | Yes  No |
|  | Did the review authors use a comprehensive literature search strategy? | Yes  Partial Yes  No |
|  | Did the review authors perform study selection in duplicate? | Yes  No |
|  | Did the review authors perform data extraction in duplicate? | Yes  No |
|  | Did the review authors provide a list of excluded studies and justify the exclusions? | Yes  Partial Yes  No |
|  | Did the review authors describe the included studies in adequate detail? | Yes  Partial Yes  No |
|  | Did the review authors use a satisfactory technique for assessing the risk of bias (RoB) in individual studies that were included in the review?  Note: Separate criteria for Randomised Controlled Trials (RCTs) and Non-randomised studies of Interventions (NRSI) | **RCTs**  Yes  Partial Yes  No  Includes only NRSI  **NRSI**  Yes  Partial Yes  No  Includes only RCTs |
|  | Did the review authors report on the sources of funding for the studies included in the review? | Yes  No |
|  | If meta-analysis was performed did the review authors use appropriate methods for statistical combination of results?  Note: Separate criteria for Randomised Controlled Trials (RCTs) and Non-randomised studies of Interventions (NRSI) | **RCTs**  Yes  No  No meta-analysis conducted  **NRSI**  Yes  No  No meta-analysis conducted |
|  | If meta-analysis was performed did the review authors assess the potential impact of RoB in individual studies on the results of the meta-analysis or other evidence synthesis? | Yes  No  No meta-analysis conducted |
|  | Did the review authors account for RoB in individual studies when interpreting/ discussing the results of the review? | Yes  No |
|  | Did the review authors provide a satisfactory explanation for, and discussion of, any heterogeneity observed in the results of the review? | Yes  No |
|  | If they performed quantitative synthesis did the review authors carry out an adequate investigation of publication bias (small study bias) and discuss its likely impact on the results of the review? | Yes  No  No meta-analysis conducted |
|  | Did the review authors report any potential sources of conflict of interest, including any funding they received for conducting the review? | Yes  No |
| **Quality - Overall** | Overall assessment | Free text: discuss overall quality of review. |

1. If more than one intervention, tool or approach is examined within an individual study, a separate row will be included to capture details of all each intervention, tools and approaches with empirical data to report. [↑](#footnote-ref-1)
2. Based on coding tool developed by Mazerolle et al. (2020) [↑](#footnote-ref-2)
3. Based on coding tool developed by Mazerolle et al. (2020) [↑](#footnote-ref-3)
4. Based on coding tool developed by Mazerolle et al. (2020) [↑](#footnote-ref-4)
5. Based on coding tool developed by Mazerolle et al. (2020) [↑](#footnote-ref-5)
6. Based on Sterne, J. A. C., Higgins, J. P. T., Elbers, R. G., Reeves, B. C. and the development group for ROBINS- I. Risk Of Bias In Non-randomized Studies of Interventions (ROBINS-I): detailed guidance, updated 12 October 2016. Available from http://www.riskofbias.info [accessed 12^th^ April 2022]. [↑](#footnote-ref-6)
7. Based on Sterne et al. (2019). [↑](#footnote-ref-7)
8. https://www.ephpp.ca/PDF/Quality%20Assessment%20Tool_2010_2.pdf [↑](#footnote-ref-8)
9. Available at https://casp-uk.net/casp-tools-checklists/. The original tool also includes a question relating to how the value of the research that was not used to assess studies. [↑](#footnote-ref-9)
10. If more than one intervention, tool or approach examined within an individual study, a separate row will be included to capture details of each intervention, tool or approach with empirical data to report. [↑](#footnote-ref-10)
11. From Shea et al. (2017). The criteria for coding each question as Yes, Partial Yes (where relevant), or No will be based on the more detailed criteria included in the full AMSTAR 2 tool, which is available from https://www.bmj.com/content/bmj/suppl/2017/09/21/bmj.j4008.DC1/sheb036104.wf1.pdf. [↑](#footnote-ref-11)
